# Xiaoyu Xiezhuo Drink Protects against Ischemia-Reperfusion Acute Kidney Injury in Aged Mice through Inhibiting the TGF-*β*1/Smad3 and HIF1 Signaling Pathways

**DOI:** 10.1155/2021/9963732

**Published:** 2021-09-09

**Authors:** Qingqing Ye, Hongbo Chen, Hongzhen Ma, Xiaojun Xiang, Shouci Hu, Cong Xia, Lanjun Fu

**Affiliations:** Department of Nephrology, The First Affiliated Hospital of Zhejiang Chinese Medical University, Zhejiang, China

## Abstract

Acute kidney injury (AKI) is responsible for significant mortality among hospitalized patients that is especially troubling aged people. An effective self-made Chinese medicine formula, Xiaoyu Xiezhuo Drink (XXD), displayed therapeutic effects on AKI. However, the compositions and underlying mechanisms of XXD remain to be elucidated. In this study, we used the ultra-high-performance liquid chromatography method coupled with hybrid triple quadrupole time-of-flight mass spectrometry (UHPLC-Q-TOF-MS) to investigate the chemical components in XXD. Then, the absorbable components of XXD were identified based on the five principles and inputted into the SwissTargetPrediction and STITCH databases to identify the drug targets. AKI-related targets were collected from the GenCLiP 3, GeneCards, and DisGeNET databases. The crossover genes of XXD and AKI were identified for functional enrichment analysis. The protein-protein interaction (PPI) network of crossover genes was constructed, followed by the identification of hub genes. Subsequently, the effects and potential mechanisms of XXD on AKI predicted by the network pharmacology and bioinformatics analyses were experimentally validated in ischemia-reperfusion (I/R) injury-induced AKI aged mouse models. A total of 122 components in XXD were obtained; among them, 58 components were found that could be absorbed in the blood. There were 800 potential drug targets predicted from the 58 absorbable components in AKI which shared 36 crossover genes with AKI-related targets. The results of functional enrichment analysis indicated that crossover genes mostly associated with the response to oxidative stress and the HIF1 signaling pathway. In the PPI network analysis, 12 hub genes were identified, including ALB, IL-6, TNF, TP53, VEGFA, PTGS2, TLR4, NOS3, EGFR, PPARG, HIF1A, and HMOX1. In AKI aged mice, XXD prominently alleviated I/R injury-induced renal dysfunction, abnormal renal pathological changes, and cellular senescence, inflammation, and oxidative damage with a reduction in the expression level of the inflammatory mediator, *α*-SMA, collagen-1, F4/80, TP53, VEGFA, PTGS2, TLR4, NOS3, EGFR, PPARG, HIF1A, ICAM-1, TGF-*β*1, Smad3, and p-Smad3 and an increase of nephridial tissue p-H3, Ki67, HMOX1, MMP-9, and Smad7 levels. In summary, our findings suggest that XXD has renoprotective effects against AKI in aged mice via inhibiting the TGF-*β*1/Smad3 and HIF1 signaling pathways.

## 1. Introduction

Acute kidney injury (AKI), previously called acute renal failure (ARF), is a serious clinical disease characterized by a rapid increase in serum creatinine and a rapid decline in glomerular filtration rate (GFR) [1, 2]. Because of an aging population, it can be seen in up to 7% of hospital admissions and 30% of ICU admissions [3]. Moreover, it has been demonstrated that AKI leads to the accumulation of water, sodium, several electrolyte disturbances, renal fibrosis, and, eventually, chronic kidney disease (CKD) [3, 4]. Significantly, the long-term prognosis of AKI, especially in the elderly, is not optimistic. Compared with younger people, a higher incidence of AKI was observed in older adults [5, 6]. Additionally, the epidemiological study of AKI in China showed that persons over 60 years old were accounted for 57.73% among 7604 AKI patients [7]. Thus, novel effective treatment strategies for AKI in older patients are still desired.

Ischemia is considered as the main cause that contributes to the development of AKI [8]. Ischemia-reperfusion (I/R) injury is tissue damage that can result from blood reperfusion, and I/R injury of kidney tissue is a common reason for AKI progression [2, 8]. Besides, the pathophysiology of AKI to CKD transition is complex, and many factors such as cell senescence, apoptosis, inflammatory processes, and oxidative stress are involved [9]. Aging is a process in which the cell structure and function of various tissues and organs in the body undergo decay and change with age under the influence of long-term internal and external environments [10]. Also, aging leads to the reduction of the kidney's resistance to (I/R) injury, drugs, contrast agents, and other poisons, so the elderly are more likely to develop AKI [11]. Renal fibrosis is the main manifestation of AKI and renal cell senescence. The process of cell senescence is accompanied by a series of changes in gene and protein expression in the aging kidney. I/R injury also exaggerates metabolic imbalance that leads to inflammation with the upregulation of proinflammatory mediators including interleukin 6 (IL-6), tumor necrosis factor *α* (TNF-*α*), intercellular adhesion molecule (ICAM-1), transforming growth factor *β* (TGF-*β*), and metalloproteinase 7/9 (MMP-7/9) [12, 13]. Furthermore, the p53/p21 pathway was activated in the renal tubular cells under the condition of acute stress, which blocks the cell cycle at the G1/S phase [14, 15]. Therefore, these studies suggested that I/R injury and renal fibrosis might serve as potential therapeutic targets for AKI in elderly men.

Traditional Chinese medicine (TCM) has attracted wide attention for the clinical treatment of AKI [16]. For instance, the ethanol extract of root bark of Illicium henryi was significantly dose-dependently against AKI in mice by inhibiting inflammatory responses and oxidative stress [17]. Xiaoyu Xiezhuo Drink (XXD) consists of Astragalus membranaceus (15 g), processed Radix cyathulae (6 g), Peach Kernel (6 g), Lumbricus (6 g), prepared Rhubarb (5 g), and Plantain (10 g). It is derived from the “Buyang Huanwu Decoction” in the book of “Yilin Gaicuo” by Wang Qingren in the Qing Dynasty. Previous studies have shown that XXD is effective in treating patients with AKI. Animal studies have shown that XXD can alleviate pathological damage, reduce the protein expression levels of *α*-smooth muscle actin (*α*-SMA), TGF-*β*1, and collagen-1, as well as upregulate Smad7 levels in the kidney tissues of mice with unilateral ureteral occlusion (UUO) [18, 19]. Taken together, these data indicated that XXD treatment markedly suppressed renal interstitial fibrosis by regulating the TGF-*β*/Smad singling pathway. However, the mechanism of XXD in the improvement of the aging kidney with I/R injury is unclear.

In recent years, the network pharmacology, first proposed by Hopkins in 2007 [20], was used as a promising strategy to investigate TCM from a system perspective based on system biology, bioinformatics, and high-throughput histology [21]. XXD is composed of many herbs, which result in a complex chemical composition. Therefore, it is difficult to fully understand their therapeutic mechanisms. Thus, it is necessary to extend the understanding of the antifibrosis effects of XXD in treating AKI of the elderly with I/R injury by using scientific and technologic approaches. In the present study, the components of the XXD were identified with ultra-high-performance liquid chromatography coupled with UHPLC-Q-TOF-MS analysis. Then, network pharmacology was used herein to predict the targets of XXD and AKI. Finally, corresponding experiments were then performed to confirm the protective effects of XXD and verify the results of prediction in aged mice with AKI. Our study is the first to identify potential absorbable compounds in XXD and elucidate their mechanisms in AKI treatment by using the network pharmacology approach.

## 2. Material and Methods

### 2.1. Preparation of XXD Aqueous Extracts

The XXD aqueous extracts were provided by the dispensary TCM of the First Affiliated Hospital of Zhejiang Chinese Medical University (Zhejiang, China). Briefly, 48 g of dried herbs of XXD was boiled with 1000 mL of distilled water. The mixture was gradually heated to 100°C over a period of 60 min. And, the extracts were concentrated to 125 mL under reduced pressure in a rotavapor at 45°C to obtain an extract with a final concentration of 384 mg/mL, according to the weight of initially dried herbs.

### 2.2. UHPLC-Q-TOF-MS Analysis

A Waters ACQUITY I-Class Plus UPLC system (Waters Corporation, Milford, MA, USA) together with a SCIEX X-500R mass spectrometer (AB SCIEX, USA) was used for UHPLC-Q-TOF-MS analysis. Briefly, the above-mentioned 1.0 mL sample was well mixed in 1.0 mL analytical methanol. The mixed solutions were centrifuged at 14,000 rpm for 20 min, and then, the supernatant was collected before analysis. Chromatographic separations were performed on an ACQUITY UPLC BEH C18 (100 mm × 2.1mm, 1.7 *μ*m). The mobile phase consisted of acetonitrile containing 0.1% formic acid (A) and 0.1% formic acid (B). Also, the gradient elution procedures were as follows: 0~21 min, 99%~50% A; 21~25.5 min, 50%~15% A; and 25.5~27 min, 15%~1% A. The flow rate was set to 0.3 mL/min, and the injection volume was 2 *μ*L. The autosampler was maintained at 8°C. A TurboIonSpray electrospray ionization (ESI) was used in positive and negative ion scanning modes. The operating parameters were optimized as follows: ion source gas 1 (Gas1): 45, ion source gas 2 (Gas2): 55, curtain gas (CUR): 35, source temperature: 600°C, IonSapary Voltage Floating (ISVF): 5500 V (ESI^+^) or -4500 V(ESI^−^); TOF MS scan at a range of 100-1500 Da, production scan at a range of 25-1500 Da, TOF MS scan accumulation time: 0.25 s/spectra, and product ion scan accumulation time: 0.035 s/spectra. Secondary mass spectrometry was obtained by using information-dependent acquisition (IDA) with a high sensitivity mode. The parameters of IDA were optimized as follows: collision energy: 35 ± 15 eV, exclude isotopes within 4 Da, and candidate ions to monitor per cycle: 12. The accurate mass and elemental composition for the precursor ions and fragment ions were analyzed by the SCIEX OS software which has a self-contained TCM MS/MS library.

### 2.3. Identification of Absorbable Components of XXD

First, the molecular formulas of the chemical components were retrieved from the PubChem (https://pubchem.ncbi.nlm.nih.gov/) database that quickly finds chemical information from authoritative sources [22], and the canonical SMILES (simplified molecular-input line-entry system) format of a specific structural formula was acquired. Then, the Molinspiration SMILES online platform (https://www.molinspiration.com/) was employed to calculate the molecular properties and bioactivity score by using the SMILES format of the chemical components. When a chemical component met the basic requirements of the related parameters, based on the five principles of drug absorption, it was considered to be the absorbable component: relative molecular mass MW ≤ 500, hydrogen bond acceptor (the number of O and N) nON ≤ 10, hydrogen bond donor (the number of hydrogen atoms attached to the O and N) nOHNH ≤ 5, and fat water partition coefficient miLogP ≤ 5.

### 2.4. Prediction of Drug Targets for XXD

The potential protein targets of the absorbable components in XXD were retrieved from a web tool, SwissTargetPrediction (http://www.swisstargetprediction.ch/), wherein the predictions are based on the similarity principle, through reverse screening [23], and the STITCH database (version 5.0, http://stitch.embl.de/) with the species selected as “*H. sapiens*” [24]. Only the protein targets which had direct interactions with each absorbable component in XXD were saved as the potential targets. After integration of potential protein targets, the absorbable component-target network was generated using Cytoscape software (version 3.6.1, https://cytoscape.org/, Boston, MA, USA) [25].

### 2.5. Collection of AKI-Related Targets

Related targets of AKI were collected from the GenCLiP 3 (http://ci.smu.edu.cn/genclip3/analysis.php) [26], GeneCards (version 5.0, https://www.genecards.org/) [27], and DisGeNET (version 7.0, http://www.disgenet.org/) [28] databases with the keyword “Acute kidney injury.”

### 2.6. Functional Enrichment and Protein-Protein Interaction (PPI) Network Analysis

Based on the previous steps, 4 sets of target lists were prepared. Then, we matched the potential drug targets of XXD absorbable components and the AKI-related targets using the Venny 2.1 (https://bioinfogp.cnb.csic.es/tools/venny/index.html); then, the crossover genes were chosen as the potential targets of XXD in treating AKI. The Metascape (http://metascape.org/gp/index.html#/main/step1), a gene annotation and analysis interactive platform [29], was used for Gene Ontology- (GO-) biological process (BP) enrichment and Kyoto Encyclopedia of Genes and Genomes (KEGG) pathway enrichment analyses on the crossover genes. To further investigate the pharmacological mechanisms of XXD on AKI, the crossover genes were then processed by the STRING database to construct a PPI network (version 11.0, https://string-db.org/) [30], with species limited to “*H. sapiens*” and a confidence score ≥ 0.7 and visualized by Cytoscape. Additionally, topological features of the PPI network were analyzed using the plug-in NetworkAnalyzer. The node size was visualized by the degree value, meaning the number of combined crossover genes, and the degree greater than the median degree of all nodes was selected as the core target for treating AKI.

### 2.7. Experimental Animals and Treatment

Fifty healthy eighteen- to twenty-month-old male C57BL/6 mice (approximately 27~32 g) were purchased from Vital River Laboratory Animal Co., Ltd. (License number: SCXK (Beijing) 2012-0001; Beijing, China) and housed under standard conditions with a 12 h light-dark cycle, humidity of 50% ± 10%, and temperature of 24 ± 1°C at the Animal Center of Zhejiang Chinese Medicine University. All experimental procedures were approved by the Zhejiang Chinese Medical University and were conducted according to the Guide for the Care and Use of Laboratory Animal of the National Institute of Health (Publication No. 80-23, revised 1996). All mice had free access to food and water during the experiments. Also, mice were acclimatized for 1 week before use. According to previously published methods by Arfian et al. [13], bilateral renal pedicle clamping was conducted to induce kidney I/R injury in this study. Briefly, the mice were intraperitoneally injected with sodium pentobarbital (0.1 mL/10 g body weight). After being anesthetized, the abdomen was opened. Then, both renal pedicles were exposed and clamped with a nontraumatic vascular clamp for 30 minutes, which was then removed. After the operating day, the mice were weighed and divided into five groups (*n* = 10): the control group, the model group, and the XXD groups (7.28 g/kg, 14.56 g/kg, and 29.12 g/kg). Besides, the sham operation procedure was used for the control group with only opening of the abdomen of the mice without renal pedicle clamping. The mice in the XXD groups were administrated with XXD at the doses of 7.28 g raw herbs/kg, 14.56 g raw herbs/kg, and 29.12 g raw herbs/kg (0.1 mL/10 g of mouse weight) by oral gavage every day for 14 days. The dosage for mice was 7.28 g raw herbs/kg according to the human mouse equivalent dosage conversion of human clinical dosage (48 g/60 kg/day). The mice in the control and model groups were given an equal volume of 0.9% physiological saline. At the end of the experiment, the urine was collected. After the final administration for 2 hours, all mice were weighed and anesthetized by sodium pentobarbital. Then, the blood samples were collected from the eyes, and the serum was obtained by centrifuging at 3000 rpm for 15 min at 4°C. Subsequently, all the mice were sacrificed via cervical dislocation, and the renal tissues were removed, weighed, and stored at -80°C for subsequent analyses. Furthermore, the renal index was calculated by using the following formula: kidney weight/mouse body weight × 100 (g/100 g body weight).

### 2.8. Liver and Renal Function Assessment

Liver function was assessed by serum aspartate aminotransferase (AST) and alanine aminotransferase (ALT). Meanwhile, renal function was evaluated by serum creatinine (Cre), blood urea nitrogen (BUN), and urinary albumin (ALB) on a Hitachi 7180 automatic biochemical analyzer (Kyoto, Japan).

### 2.9. Histology Assessment and SA-*β*-gal Staining

To evaluate histological changes, a part of kidney tissues was fixed in 4% paraformaldehyde. After that, the samples were embedded in paraffin and cut into 4 *μ*m thick sections for hematoxylin and eosin (HE) and Masson staining for histological analysis and renal interstitial fibrosis observation, respectively. Also, SA-*β*-gal staining analysis was performed to assess the cellular senescence in kidney tissues of I/R-induced AKI aged mice with the SA-*β*-gal staining kit (ab65351, Abcam, Cambridge, MA), according to the manufacturer's protocol. Finally, the images were captured by optical microscopy (DM3000, Leica, Germany) with 200x and 400x magnifications.

### 2.10. Enzyme-Linked Immunosorbent Assay (ELISA)

Oxidative stress levels of kidney tissues were assessed using malondialdehyde (MDA), superoxide dismutase (SOD), and glutathione (GSH) activities. Briefly, renal tissue samples (100 mg) were mixed with 900 *μ*L of PBS to prepare 10% homogenate by a tissue homogenizer (Shanghai, China). Then, the homogenate supernatants were separated by centrifuging at 12,000 rpm for 20 min at 4°C. Meanwhile, the protein concentration of the supernatants was quantified using the BCA Protein Assay Kit (Beyotime, China). The collected supernatants were measured for renal MDA, SOD, and GSH levels using commercial kits (Nanjing Jiancheng Bioengineering Institute, Nanjing, Jiangsu, China) based on the manufacturer's instruction.

### 2.11. Immunohistochemistry

The expression of *α*-SMA, collagen-1, classic renal macrophage marker (F4/80), phosphohistone H3 (p-H3), and proliferation marker (Ki67) was assessed by immunohistochemical staining. Briefly, 4 sections were deparaffinized, and endogenous peroxidase activity was blocked by incubating in 3% hydrogen peroxide at 37°C for 30 min. Then, the sections were incubated with 5% BSA for 30 min at 37°C to block the nonspecific antibody binding. Primary antibody of *α*-SMA (1 : 500, AF1032, Affinity Biosciences, USA), collagen-1 (1 : 500, ab270993, Abcam, USA), F4/80 (200 *μ*g/mL, sc-377009, Santa Cruz Biotechnology (Shanghai) Co., Ltd., USA), p-H3 (1 : 300, H6409, MilliporeSigma, Germany), and Ki67 (5 *μ*g/mL, ab15580, Abcam, USA) was applied to the sections and incubated overnight at 4°C. After washing three times with PBS, the sections were incubated with biotinylated goat anti-mouse/rabbit IgG (0.5 *μ*g/mL; Abcam) for 1 h at room temperature and stained using 3,3′-diaminobenzidine dihydrochloride. Digital images of immunohistochemistry staining were captured with the light microscope and *α*-SMA, collagen-1, F4/80, p-H3, and Ki67 expressions were quantified with ImageJ software (National Institutes of Health, NY).

### 2.12. Quantitative Real-Time Polymerase Chain Reaction (PCR) Analysis

Total RNAs were isolated from renal tissues using a TRIzol Reagent (Invitrogen) and quantified by a NanoDrop 2000/2000c spectrophotometer (Thermo Fisher Scientific, Waltham, MA, USA). The complementary DNA (cDNA) was synthesized with the 1 *μ*g total RNA and a reverse transcription kit (TaKaRa, Tokyo, Japan). The quantitative real-time PCR was performed using the SYBR® Premix Ex Taq™ II reagent kit (TaKaRa) and specific primers on a CFX96 real-time PCR system (Bio-Rad, USA). The PCR amplification conditions were 95°C for 5 min, 95°C for 10 s, 55°C for 30 s, and 72°C for 10 s, for 40 cycles. The primers were synthesized by Sangon (Shanghai, China) and listed in Table 1. Gene expression was normalized to GAPDH as internal controls and calculated using the 2^−*ΔΔ*Ct^ method.

### 2.13. Western Blotting

The renal tissues were homogenized in a cold RIPA lysis buffer (Thermo Fisher Scientific) including protease and phosphatase inhibitors (Sigma-Aldrich). Then, the total protein was extracted after being centrifuged at 12,000 rpm for 15 min at 4°C. The protein concentration was measured with the BCA method. After that, the protein was denatured by storing in the water of 100°C for 5 min after being mixed with the loading buffer, and the supernatant was used for western blotting analysis. Protein samples were separated by 10% serum dodecyl sulfate-polyacrylamide gels (SDS-PAGE) and then transferred to polyvinylidene fluoride (PVDF) membranes (Merck Millipore, USA). The membranes were blocked with 5% nonfat milk and incubated with primary antibodies against MMP-9 (1 : 2000, ab76003, Abcam, Cambridge, UK), *α*-SMA (1 : 1000, AF1032, Affinity Biosciences, USA), ICAM-1 (1 : 2000, BF0097, Affinity Biosciences, USA), TGF-*β*1 (1 : 1000, ab215715, Abcam), Smad7 (1 : 1000, ab216428, Abcam), HIF1A (1 : 2000, BF0593, Affinity Biosciences, USA), total Smad3 (1 : 1000, ab40854, Abcam), p-Smad3 (1 : 2000, ab52903, Abcam), GAPDH (1 : 5000, D190090, Sangon, Shanghai, China), and *β*-actin (1 : 5000, ab8227, Abcam) for a whole night at 4°C, followed by incubation with goat anti-rabbit antibody (ab205718, 1 : 5000) and goat anti-mouse (ab205719, 1 : 5000) for 1 h at room temperature. GAPDH was used as an internal control. The blots were visualized with an ECL kit (P0018S, Beyotime, Beijing, China), and the protein intensity was quantified with ImageJ software.

### 2.14. Statistical Analysis

All the data were presented as mean ± standard deviation (SD). Statistical analysis was performed by SPSS software (version 21.0; SPSS Inc., Chicago, IL, USA). Student's *t*-test was applied to the analysis of two groups, and a comparison between multiple groups was done using one-way ANOVA. *P* < 0.05 is considered statistically significant.

## 3. Results

### 3.1. Identification of Absorbable Components in XXD Aqueous Extract

**Figure 1:**
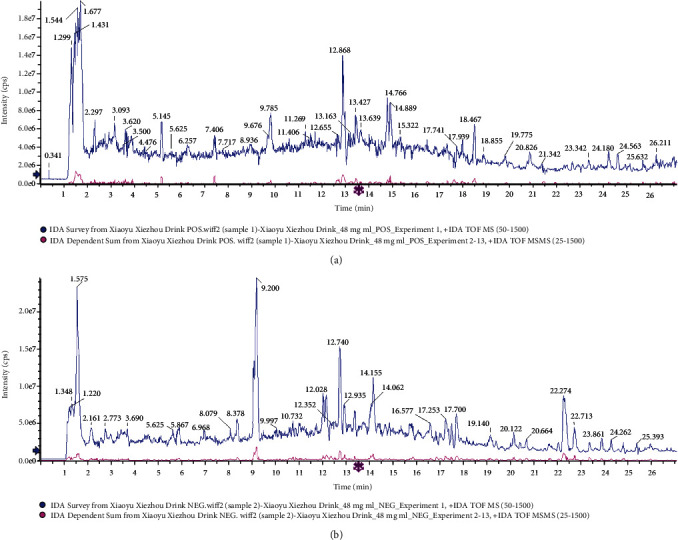
The base peak chromatograms of the XXD extract by UPLC-Q-TOF-MS analysis in positive (a) and negative (b) ion modes.

In this study, the phytochemical compositions of XXD were identified using UHPLC-Q-TOF-MS analysis based on the TCM MS/MS Library in SCIEX OS software. The base peak chromatograms of the XXD aqueous extract in positive and negative ion modes are presented in Figure 1. The identification results for the constituents in XXD are presented in Table S1 and Table S2. Fifty-five compounds were identified under the positive ion mode and sixty-seven compounds were identified under the negative ion mode. Using the Molinspiration to calculate the identified compounds of XXD, we obtained absorption parameters that could estimate whether the chemical compositions could be absorbed. As a result, Table 2 indicated that there were a total of 58 components that met the five principles of drug absorption after eliminating the redundancy.

### 3.2. Absorbable Component-Target Network Analysis

**Figure 2:**
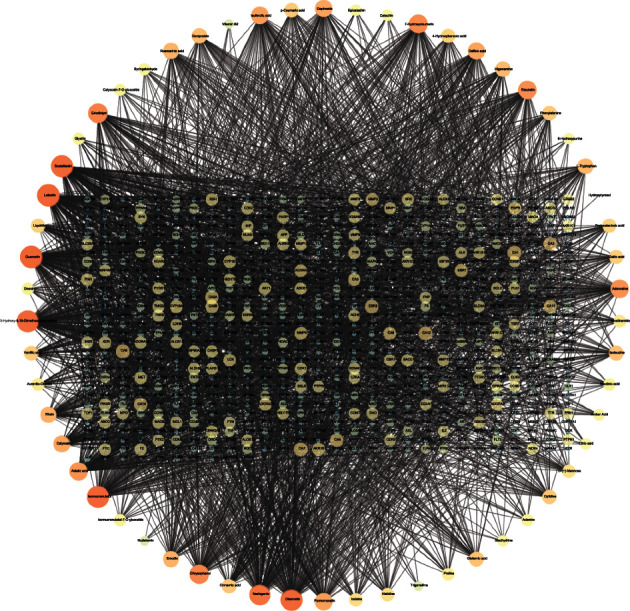
The compound-target network for XXD. The nodes in circles represent candidate absorbable compounds, and nodes with the rectangular arrangement represent potential protein targets. The edges represent the interactions between them. Node size and color are visualized by using their degree.

Among the 58 candidate bioactive components, 2622 protein targets were retrieved from the SwissTargetPrediction and STITCH databases. After eliminating the overlaps, 800 drug-protein targets of XXD were obtained for further analyses. As shown in Figure 2, There are 858 nodes (800 compound target nodes and 58 compound nodes) and 2620 edges which composed the absorbable component-target network. In this network, we found some special absorbable compounds with a higher degree interacting with multiple targets such as luteolin (degree = 114), quercetin (degree = 114), isomucronulatol (degree = 111), 3-hydroxy-9,10-dimethoxypterocarpan (degree = 110), scutellarein (degree = 109), diosmetin (degree = 109), and naringenin (degree = 102). These high-degree absorbable components in the network may account for the essential therapeutic effects of XXD on AKI.

### 3.3. Target Identification and Analysis

**Figure 3:**
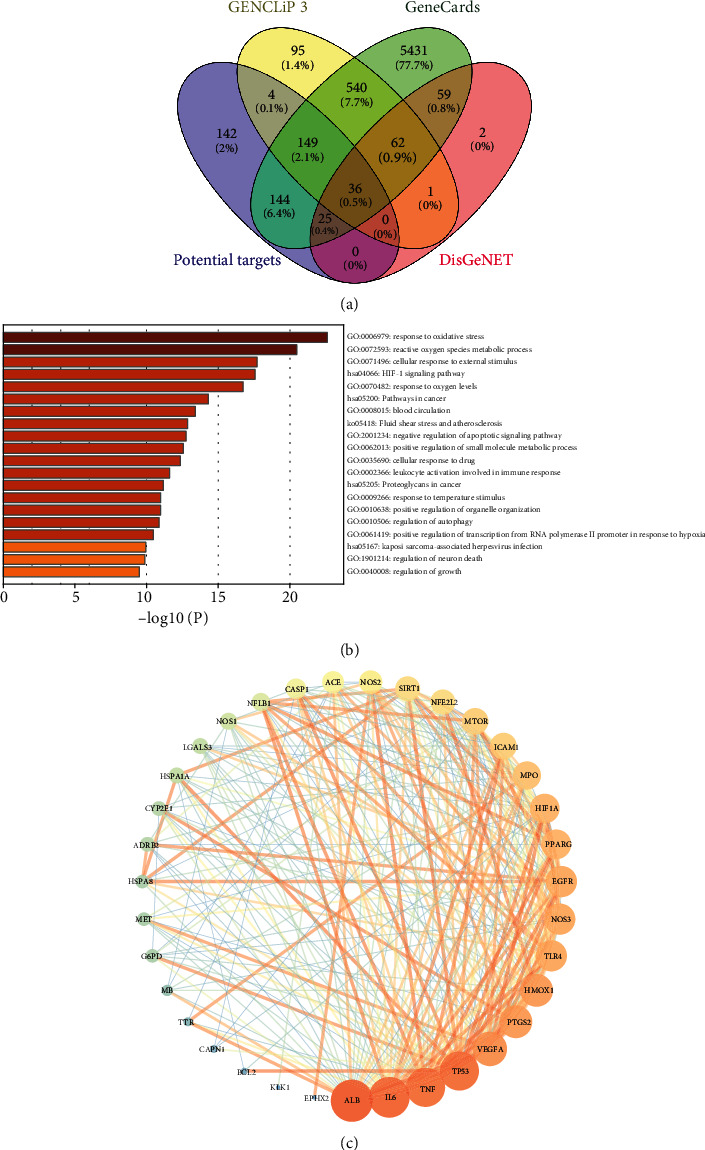
Target identification of XXD on AKI and analysis. (a) The Venn diagram of the targets in both XXD targets and AKI targets. (b) The GO-BP and KEGG pathway enrichment analyses of 36 involved targets in XXD. (c) The PPI network analysis of 35 potential targets. Node size and color are proportional to the target degree in the network.

In total, 887 AKI-related genes were collected from the GenCLip 3 database. 6746 AKI-related genes were collected from the GeneCards database. And 185 AKI-related genes were acquired based on the DisGeNET database. Then, drug targets of XXD were mapped with AKI-related genes. As a result, 36 crossover genes of 58 components in XXD were associated with AKI and are shown in Figure 3(a) such as TLR4, ACE, HSPA8, SIRT1, EGFR, HSPA1A, HMOX1, NOS2, NOS1, NOS3, ADRB2, PTGS2, MET, IL-6, CYP2E1, LGALS3, EPHX2, TTR, TNF, SLC22A12, MPO, MTOR, VEGFA, TP53, BCL2, CAPN1, PPARG, KLK1, MB, ALB, CASP1, ICAM-1, NFE2L2, NFKB1, HIF1A, and G6PD.

To illustrate the biological characteristics of AKI-related genes of XXD on AKI, we performed GO and KEGG pathway enrichment analyses of the 36 involved targets via the Metascape database. The detailed GO terms and pathway information of XXD on AKI are shown in Figure 3(b). As a result, the targets of XXD were enriched in response to oxidative stress (GO:0006979), reactive oxygen species metabolic process (GO: 0072593), and cellular response to external stimulus (GO: 0071496), etc. Meanwhile, we observed that the HIF1 signaling pathway (hsa04066), proteoglycans in cancer (hsa05205), and Kaposi sarcoma-associated herpesvirus infection (hsa05167) were mainly involved with the 36 targets of 58 components in XXD. Based on those GO and KEGG pathway enrichment analyses, it is possible that the anti-AKI effect of XXD results from a complex multibiological process and multipathway synergetic effect.

To study the interaction of the 36 targets in vivo and search for the hub genes, PPI network analysis was carried out. There were 35 nodes (SLC22A12, the discrete node, was removed) and 273 edges in total. The topological feature analysis of the PPI is based on the major parameters of “degree.” In this network, we used the median value of node degree (20) as a cutoff point primarily. Eventually, 12 major hubs were picked out as hub genes for further study. These genes were ALB (degree = 29), IL-6 (degree = 28), TNF (degree = 27), TP53 (degree = 27), VEGFA (degree = 24), PTGS2 (degree = 23), HMOX1 (degree = 23), NOS3 (degree = 22), TLR4 (degree = 22), EGFR (degree = 22), PPARG (degree = 21), and HIF1A (degree = 21) (Figure 3(c)).

### 3.4. XXD Ameliorated Kidney Function in AKI Mice

**Figure 4:**
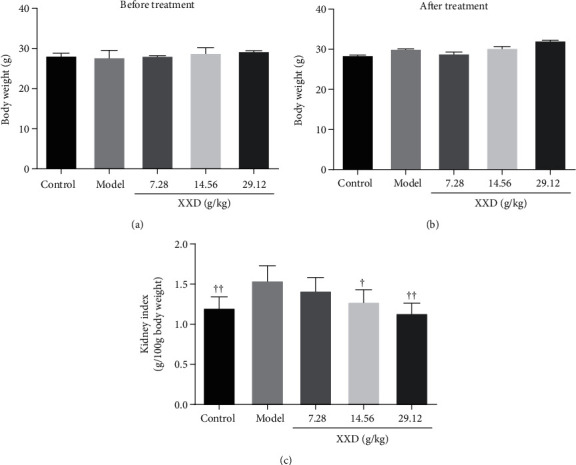
Effect of XXD on body weight of AKI mice. (a) The body weight of mice before XXD treatment. (b) The body weight of mice after XXD treatment. (d) The kidney index was calculated via kidney weight/body weight. *n* = 10 mice per group. All data are expressed as means ± SD. ^▲^*P* < 0.05, ^▲▲^*P* < 0.01 vs. the model group.

**Figure 5:**
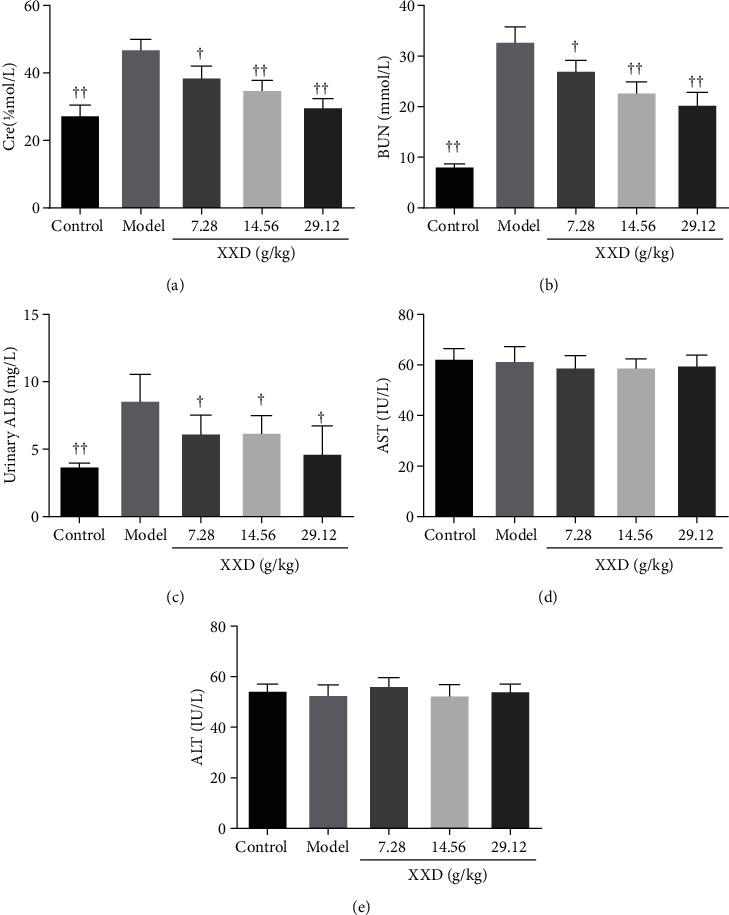
Effect of XXD on liver and kidney function of AKI mice: (a) serum creatinine (Cre), (b) blood urea nitrogen (BUN), (c) urinary albumin (ALB), (d) serum aspartate aminotransferase (AST), and (e) alanine aminotransferase (ALT). *n* = 10 mice per group. All data are expressed as means ± SD. ^▲^*P* < 0.05, ^▲▲^*P* < 0.01 vs. the model group.

To investigate the effect of XXD on aging mice with AKI, liver and kidney function indicators were evaluated. As shown in Figures 4(a) and 4(b), the body weight of AKI mice has maintained a relatively stable status after XXD treatment. They did not differ significantly compared with the model group until 14 days. Compared with the control group, the kidney index in the model group was significantly increased; however, that level in the XXD groups (14.56 and 29.12 g/kg) was significantly lower than that in the model group (Figure 4(c)). Further, we found that treatment with XXD significantly lowered serum Cre (Figure 5(a)) and BUN (Figure 5(b)) levels, which were increased in the model group, in a relatively dose-dependent manner. Additionally, the urinary ALB level in the XXD-treated group was significantly lower than that in the model group (Figure 5(c)). Notably, there was no significant difference in AST (Figure 5(d)) and ALT (Figure 5(e)) levels in aging AKI mice treated with XXD.

### 3.5. XXD Alleviated I/R-Induced Kidney Injury of Aging Mice

**Figure 6:**
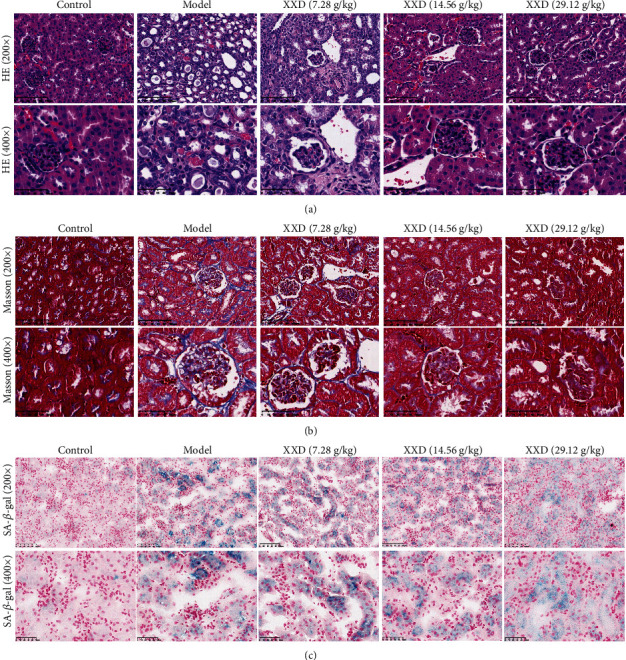
Effect of XXD on renal histopathology of the kidneys. (a) Hematoxylin and eosin (HE) and (b) Masson staining show changes in the control and model mice with XXD treatment. Bars represent 100 *μ*m and 50 *μ*m. (c) SA-*β*-gal staining of kidney tissue in AKI mice. Semiquantitative analysis of SA-*β*-gal staining showed a significant decrease of SA-*β*-gal activity in the XXD-treated kidneys as compared with the model mice.

The kidney tissues were collected and stained with H&E and Masson to explore the histopathological changes and the degree of renal fibrosis, respectively. As shown in Figure 6(a), the H&E staining results showed that the renal tissue structure was tightly arranged, the structure of the glomerulus was clear, and the kidney tubules were complete and tightly packed in the control group. In contrast to the control group, the glomerular basement membranes of the glomerular were significantly thickened, cells around the glomerulus were disordered accompanied with glomerular hypertrophy, and the kidney tubules exhibited diffuse expansion. These pathological changes were improved under XXD treatment, compared with those in the model group. Masson staining showed aging mice with AKI revealed moderate renal interstitial fibrosis. The collagen fibers in the kidney tissue in each XXD-treated group, particularly at the XXD (29.12 g/kg) group, were lower than that in the model group (Figure 6(b)). SA-*β*-gal is a common biomarker of senescence. In this study, the results showed that SA-*β*-gal activity was significantly lower in the XXD-treated kidneys than in the model groups (Figure 6(c)). Therefore, these results suggested that XXD could suppress the cellular senescence of kidney tissues in AKI aged mice.

### 3.6. XXD Suppressed Inflammation and Oxidative Stress in Kidney Tissue of AKI Mice

**Figure 7:**
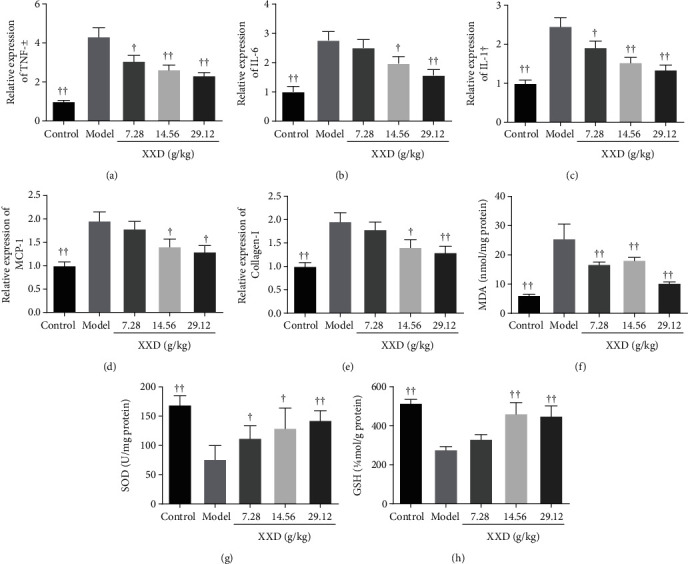
Effect of XXD on inflammation and oxidative stress in kidney tissue of AKI mice. The mRNA expression levels of (a) TNF-*α*, (b) IL-6, (c) IL-1*β*, (d) MCP-1, and (e) collagen-1 were measured using a qRT-PCR assay. The levels of (f) MDA, (g) SOD, and (h) GSH were detected using ELISA kits. Data are presented as the means ± SD (*n* = 10). ^▲^*P* < 0.05, ^▲▲^*P* < 0.01 vs. the model group.

To elucidate the effect of the XXD on inflammation and oxidative stress in kidney tissue of AKI mice, we evaluated the expression levels of TNF-*α*, IL-6, IL-1*β*, MCP-1, MDA, SOD, and GSH. As shown in Figures 7(a)–7(d), XXD significantly reduced the expression of TNF-*α*, IL-6, IL-1*β*, and MCP-1 in the kidney tissue of AKI mice. Moreover, we also found that the mRNA levels of collagen-1 were significantly decreased in XXD-treated groups (Figure 7(e)). Interestingly, XXD markedly inhibited the expression of MDA (Figure 7(f)); also, SOD and GSH levels were also increased in the kidney tissue of AKI mice upon XXD treatment (Figures 7(g) and 7(h)). These findings indicated the efficacy of XXD in protecting against AKI through inhibiting inflammation and oxidative stress.

### 3.7. XXD Downregulated *α*-SMA, Collagen-1, and F4/80 and Upregulated p-H3 and Ki67 Expression in Mice with AKI

**Figure 8:**
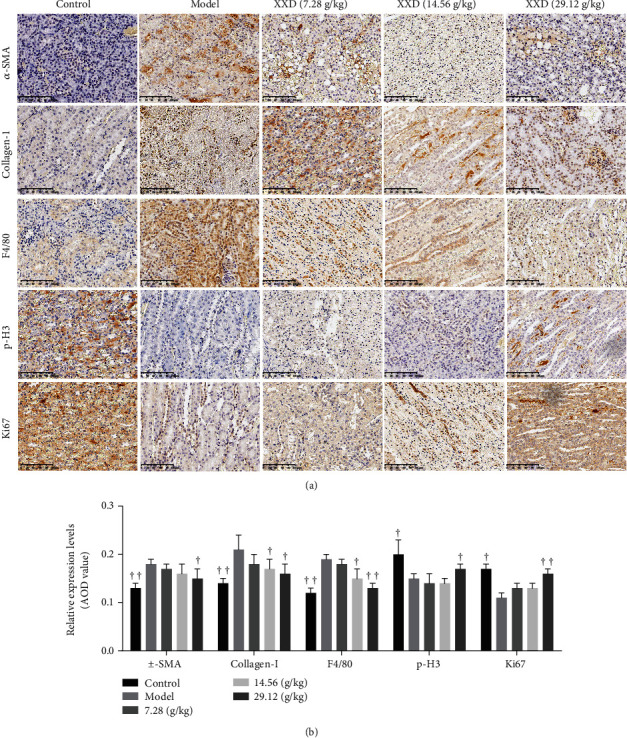
Effect of XXD on the renal fibrosis and tubular repair of AKI mice. (a) Representative immunohistochemical staining and (b) quantitative analysis of the expression of *α*-SMA, collagen-1, F4/80, p-H3, and Ki67 in XXD groups as indicated. *n* = 10 mice per group. All data are expressed as means ± SD. ^▲^*P* < 0.05, ^▲▲^*P* < 0.01 vs. the model group.

To further investigate the protective effect of XXD on the renal fibrosis of AKI mice, the expression levels of fibrotic factors, such as *α*-SMA and collagen-1, are observed in Figures 8(a) and 8(b). Compared with the control group, the expressions of *α*-SMA and collagen-1 were significantly increased in kidneys of AKI mice. However, treatment with high-dose XXD effectively reversed the increase of *α*-SMA and collagen-1. Furthermore, the presence in kidney tissue of the macrophage marker F4/80 intensively supports an inflammatory response to AKI after I/R injury. Compared to model mice, the expressions of F4/80 were significantly decreased after XXD treatment. To confirm the tubular repair induced by XXD, the expression levels of p-H3 and Ki67 were also detected by using immunohistochemistry staining. The expression of p-H3 was during the M phase of the cell cycle, and we found that the levels of p-H3 were obviously increased in the high-dose XXD group compared with model mice. Also, the immunohistochemical staining demonstrated that the expression of Ki67, a well-accepted marker of cellular proliferation, was increased in the high-dose XXD group compared with model mice. These findings suggest that the protective effect of XXD on AKI partly results from its antifibrosis, anti-inflammatory, and tubular repair effects.

### 3.8. Validation of the Hub Genes

**Figure 9:**
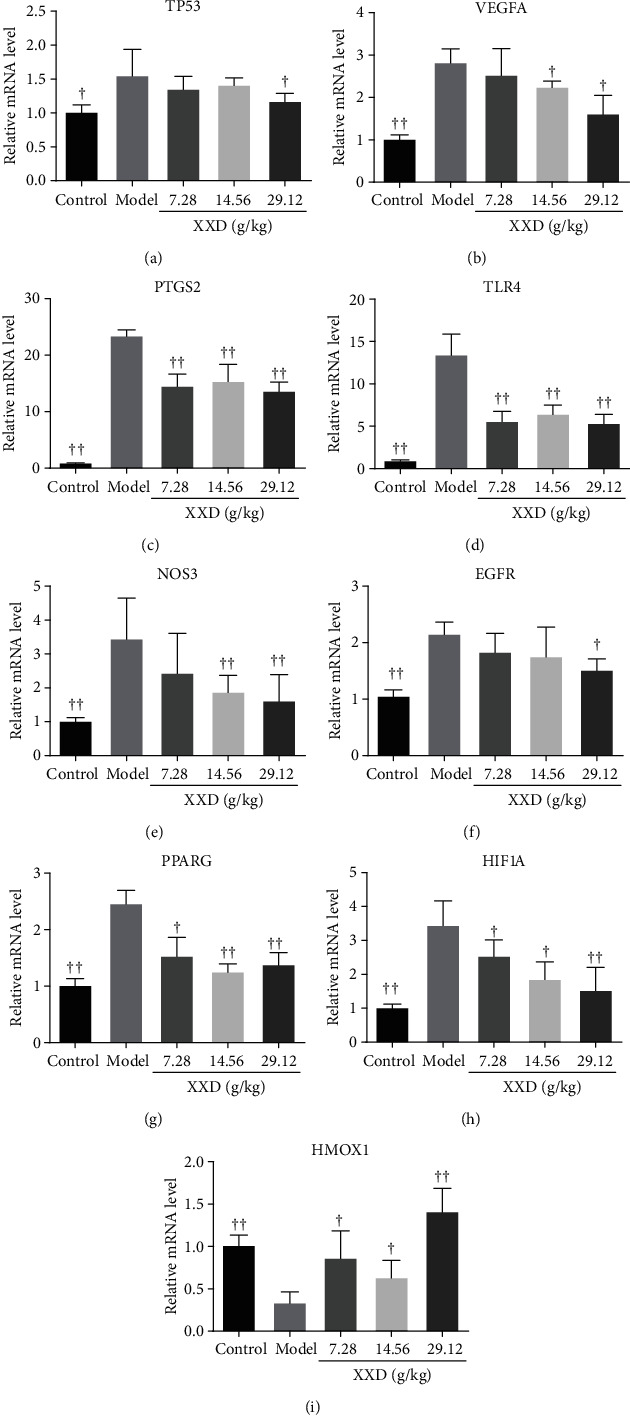
Effect of XXD on the expression levels of hub genes of AKI mice. Quantitative analysis of the expression of (a) TP53, (b) VEGFA, (c) PTGS2, (d) TLR4, (e) NOS3, (f) EGFR, (g) PPARG, (h) HIF1A, and (i) HMOX1. All experiments were performed in triplicate, and results were presented as means ± SD (*n* = 10). ^▲^*P* < 0.05, ^▲▲^*P* < 0.01 vs. the model group.

The results of bioinformatics analysis were further confirmed by using qRT-PCR on the mRNA levels of the nine hub genes in kidneys of AKI mice which were XXD-treated. Then, to a certain extent, the qRT-PCR assay showed that the relative expression levels of TP53, VEGFA, PTGS2, TLR4, NOS3, EGFR, PPARG, and HIF1A were significantly decreased in renal tissues of XXD-treated groups (Figures 9(a)–9(h)). Meanwhile, the HMOX1 levels in I/R mouse models treated with XXD were dramatically elevated by XXD (Figure 9(i)).

### 3.9. XXD Suppressed TGF-*β*1/Smad3 and HIF1 Signaling Pathways in the Kidneys of I/R AKI Mice

**Figure 10:**
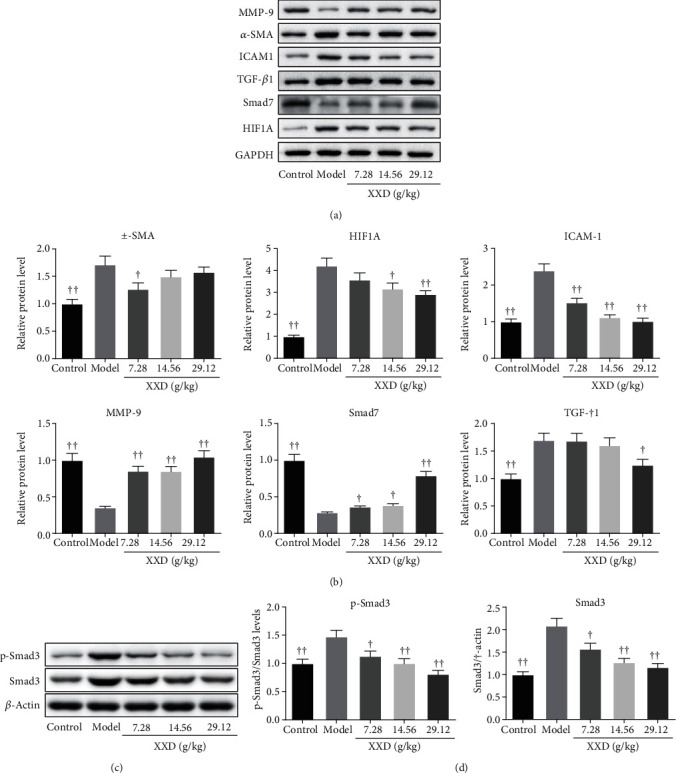
Effect of XXD on the TGF-*β*1/Smad3 and HIF1 signaling pathways of kidney tissues in AKI mice. (a) Representative western blots for MMP-9, *α*-SMA, ICAM-1, TGF-*β*1, Smad7, HIF1A, and GAPDH. Quantitative analysis of (b) MMP-9, *α*-SMA, ICAM-1, TGF-*β*1, Smad7, and HIF1A and normalized with GAPDH. (c) Representative protein bands of p-Smad3, Smad3, and *β*-actin in XXD-treated AKI mice. (d) Quantitative analysis of p-Smad3 and Smad3 and normalized with *β*-actin. All data are represented as the mean ± SD (*n* = 3). ^▲^*P* < 0.05, ^▲▲^*P* < 0.01 vs. the model group.

To identify the antifibrosis and antioxidation mechanism of XXD in the kidneys of I/R-induced AKI mice, the TGF-*β*1/Smad3 and HIF1 signaling pathways were further investigated by western blotting analysis. XXD treatment significantly increased MMP-9 expression in mouse kidneys (Figures 10(a) and 10(b)). Also, the protein expression levels of *α*-SMA, ICAM-1, TGF-*β*1, Smad3, p-Smad3, and HIF1A in kidney tissues of AKI mice were decreased after XXD administration (Figures 10(a)–10(d)). Nevertheless, western blotting indicated that high-dose XXD significantly promoted the Smad7 levels in AKI mice (Figures 10(a) and 10(b)).

## 4. Discussion

AKI, a global public health crisis, is associated with significant morbidity, and severe or repeated AKI can transition to CKD or even end-stage renal disease (ESRD), particularly in hospitalized elderly patients [13, 31, 32]. Furthermore, it is well known that ischemia-reperfusion (I/R) injury is a major risk factor for serious clinical problems and is commonly used to investigate the pathogenesis of AKI [33, 34]. Despite significant advances having been made in exploring novel therapeutic strategies, there still remained a lack of effective therapies. Therefore, it is critical to understand more about the underlying mechanism and explore a new approach to treat the disease.

TCM has been widely used in the treatment of varied chronic diseases with multiple targets and multiple pathways. Previous studies have demonstrated the biological properties of TCM in treating AKI, including antioxidant [17], anti-inflammatory [17, 35], and antiapoptosis [36]. In the current study, we successfully identified the absorbable chemical components of Xiaoyu Xiezhuo Drink (XXD) using UHPLC-Q-TOF-MS analysis. Additionally, the network pharmacology and bioinformatics methods were used to predict component targets and potential biological events in an integral view and promote efficient drug discovery. Based on the compound-target network for XXD, these flavonoids primarily included luteolin, quercetin, isomucronulatol, 3-hydroxy-9,10-dimethoxypterocarpan, scutellarein, diosmetin, and naringenin. Luteolin has been reported to ameliorate renal injury and dysfunction in wild-type C57/Bl6 mice with ischemic AKI [37]. Quercetin, a natural flavonoid, widely exists in vegetables, fruits, and Chinese herb medicine. Wang et al. considered that quercetin can protect AKI by inhibiting ferroptosis [38]. Also, Gu et al. found that quercetin may be a promising therapeutic avenue for SARS-CoV-2 injury-induced AKI [39]. Scutellarein is a flavonoid monomer that has been demonstrated to exert various pharmacological effects including anticancer [40], anti-inflammatory [41], and antifibrosis activities [42]. Furthermore, a recent study found that diosmetin also has renoprotective effects in the sepsis-induced AKI model by activating the TUG1/Nrf2/HO-1 pathway [43]. However, as far as we know, people still know little about the effect of isomucronulatol, 3-hydroxy-9,10-dimethoxypterocarpan, and naringenin on I/R-induced AKI. Taken together, these results tell us that flavonoids play an important role in the renoprotective effects of XXD.

In this study, thirty-six crossover genes were determined for drugs and diseases using mapping analysis, which might be targeted for XXD to play an anti-AKI role. Subsequently, GO analysis results showed that the target genes associated with BP were in response to oxidative stress and the reactive oxygen species metabolic process, etc. Meanwhile, KEGG analysis results showed that the target genes were involved in numerous pathways, and the HIF1 signaling pathway was ranked first. We speculated that these BP and pathways might be the core physiological events in the treatment of AKI with XXD in elderly men. According to the topological analysis of the PPI network, we further identified 12 hub genes from the 36 crossover genes for the subsequent study including ALB, IL-6, TNF, TP53, VEGFA, PTGS2 (COX-2), TLR4, NOS3 (eNOS), EGFR, PPARG (PPAR-*γ*), HIF1A, and HMOX1 (HO-1). Among them, TP53 is found to be mutated in approximately half of all tumors and accompanied by increased expression of TP53 in kidneys of AKI mice [44, 45]. VEGF is widely expressed in the mesenchymal tissue, which promoted the development of vascellum, and possesses proangiogenic effects in renal I/R injury [46]. Furthermore, Xu et al. found that the expression of VEGFA was significantly increased in AKI patients [2]. COX-2 is positively related to the severity of the inflammatory response and then aggravates kidney tubular epithelial cell injury [47]. TLR4 is one of the members of the Toll-like receptor (TLR) family, which can induce inflammatory cytokine expression, and then lead to inflammation reaction with consequent renal injury [48]. Endothelial nitric oxide synthase (eNOS) belongs to the isozymes of nitric oxide (NO) synthase and is distributed in vascular endothelial cells in the kidney. Also, Ohkita et al. demonstrated that the ameliorating effects of the grape extract existed in wild-type mice, but not in eNOS knockout mice [49]. For the EGFR, the role of EGFR in AKI may be controversial. A recent study has shown that EGFR plays a detrimental role in I/R-induced AKI [50]. Nonetheless, other studies suggest that EGFR is responsible for the renoprotective role on the renal fibrosis of I/R injury [51, 52]. Recently, Singh et al. reported that I/R injury caused a significant decrease in the levels of PPAR-*γ* and eNOS in rat kidneys [53]. It suggests that PPAR-*γ* activation may be associated with inhibition of oxidative stress and subsequent improvement of AKI. HIF1A, an oxygen-sensitive subunit, is stable under hypoxic environments. To the best of our knowledge, there are no studies that have evaluated the effect of TCM on HIF1A in I/R-induced AKI aged mouse models. HMOX1 is a cytoprotective enzyme characterized by its antiapoptotic and anti-inflammatory properties to protect against AKI [54]. As discussed above, the roles of hub genes on the processes of AKI must be further examined.

As widely accepted, I/R injury-induced AKI is characterized by inflammation, oxidative stress, and a sharp rise of serum creatinine and BUN levels. In the current study, the nephroprotective effects of XXD were investigated using an I/R injury-induced AKI aged mouse model. Interestingly, we observed that XXD significantly decreased the serum Cre, BUN, urinary ALB, and inflammatory factor levels and enhanced the antioxidant activities of the kidney in AKI aged mice. Moreover, XXD effectively improved the histological changes, collagen deposition, and cell senescence in renal tissues of AKI mice. Our study also demonstrated XXD treatment ameliorating kidney fibrosis and promoting cellular proliferation, as accompanied by attenuating the protein expression of *α*-SMA, collagen-1, F4/80, and ICAM-1, as well as dramatically increasing the p-H3, Ki67, and MMP-9 levels. Additionally, the qPCR analysis demonstrated that the TP53, VEGFA, PTGS2, TLR4, NOS3, EGFR, PPARG, and HIF1A were downregulated, while HMOX1 was found to be upregulated in XXD-treated AKI aged mice. TGF-*β*1 has been reported to promote fibrosis in the kidney with I/R injury [13]. Furthermore, a recent study demonstrated that the knockout of Smad3 attenuated I/R-induced AKI in diabetic kidney by interacting with p53 and NOX4 [55]. Additionally, many studies have shown that overexpression of Smad7 caused the inhibition of renal inflammation by inhibiting NF-*κ*B activation [56, 57]. Smad7, a negative regulator of TGF-*β* signaling, is able to compete with Smad3, in order to inhibit the phosphorylation of receptor-activated Smads [58]. In this regard, it is worth noting the effects of XXD on the TGF-*β*/Smads in AKI aged mice. As we have shown, we also found that there was downregulation of TGF-*β*1, Smad3, Smad3 phosphorylation, and elevated Smad7 protein levels after XXD treatment in AKI aged mice. Taken together, these investigations suggested that XXD exerted its renoprotective effects via ameliorating inflammation and oxidative stress by inhibiting the TGF-*β*1/Smad3 and HIF1 signaling pathways.

## 5. Conclusion

In summary, the pharmacological mechanism of XXD to ameliorate ischemic AKI was explored with the combination of network pharmacology prediction and experimental validation. By constructing component-target and PPI networks, the flavonoids and several hub genes such as luteolin, quercetin, scutellarein, diosmetin, TP53, VEGFA, PTGS2, TLR4, NOS3, EGFR, PPARG, HIF1A, and HMOX1 might be promising for the treatment of AKI. Furthermore, We discovered that the effects of XXD may be associated with its anti-inflammatory and antioxidant activities in I/R injury-induced AKI aged mice via the regulation of TGF-*β*1/Smad3 and HIF1 signaling pathways, which provide new insights for strengthening the understanding of the therapeutic effects of XXD on AKI. However, to confirm the effects of XXD, further experimental experiments are needed to validate these results in various AKI models and large-scale clinical trials.

## Figures and Tables

**Table 1:** Primers used for qRT-PCR.

Gene name	Forward (5′~3′)	Reverse (5′~3′)
TNF-*α*	CAGGCGGTGCCTATGTCTC	CGATCACCCCGAAGTTCAGTAG
IL-6	GGCGGATCGGATGTTGTGAT	GGACCCCAGACAATCGGTTG
IL-1*β*	GAAATGCCACCTTTTGACAGTG	TGGATGCTCTCATCAGGACAG
MCP-1	CTGAGTTGACTCCTACTGTGGA	TCTTCCCAGGGTCGATAAAGT
Collagen-1	AATGGAAGTTCTACTCGCGTAGG	TTCTCGCCTGGTTGACCTTTG
TP53	ACCTATGGAAACTACTTCCTGAAA	ACCATCGCTATCTGAGCAGC
VEGFA	GGCAAAAACGAAAGCGCAAG	ATTAGACAGCAGCGGGCAC
PTGS2	GTTCCACCCGCAGTACAGAA	AGGGCTTCAGCATAAAGCGT
TLR4	ATCCCCTGAGGCATTTAGGC	GTCAAATTGCACAGGCCCTC
NOS3	GACCCACTGGTGTCCTCTTG	CTCCGTTTGGGGCTGAAGAT
EGFR	ACGAGTAACAAGCTCACGCA	GATCACACTTTTGGCCCTGT
PPARG	ACTTTGGGATCAGCTCCGTG	TGGGCGGTTGATTTGTCTGT
HIF1A	CCAATGTCGGAGTTTGGAAAACA	GTGGCAACTGATGAGCAAGC
HMOX1	GTGCCACCAAGTTCAAGCAG	CAGCTCCTGCAACTCCTCAA
GAPDH	TGTCAAGCTCATTTCCTGGTATG	TTATGGGGGTCTGGGATGGA

**Table 2:** The absorbable components of XXD aqueous extract.

No.	Component name	Formula	Mass	nON	nOHNH	miLogP	Results
1	Betaine	C5H11NO2	118.0861	3	0	-5.41	✔
2	Trigonelline	C7H7NO2	138.0551	3	0	-5.4	✔
3	Proline	C5H9NO2	116.0706	3	2	-1.72	✔
4	Stachydrine	C7H13NO2	144.1018	3	0	-5.31	✔
5	Adenine	C5H5N5	136.0616	5	3	0.23	✔
6	Cytidine	C9H13N3O5	244.093	8	5	-1.93	✔
7	Nicotinic acid	C6H5NO2	124.0391	3	1	0.27	✔
8	Nicotinamide	C6H6N2O	123.0552	3	2	-0.48	✔
9	Adenosine	C10H13N5O4	268.1038	9	5	-0.85	✔
10	6-Hydroxypurine	C5H4N4O	137.0457	5	2	-0.73	✔
11	Phenylalanine	C9H11NO2	166.0862	3	3	-1.23	✔
12	Higenamine	C16H17NO3	272.1284	4	4	2	✔
13	4-Hydroxybenzoic acid	C7H6O3	139.0389	3	2	1.37	✔
14	Catechin	C15H14O6	291.0866	6	5	1.37	✔
15	Epicatechin	C15H14O6	291.0866	6	5	1.37	✔
16	Daphnetin	C9H6O4	179.0339	4	2	1.25	✔
17	Vitamin B2	C17H20N4O6	377.1458	10	5	-0.76	✔
18	Geniposide	C17H24O10.NH3	406.1711	10	5	-1.53	✔
19	Syringaldehyde	C9H10O4	183.0652	4	1	1.08	✔
20	Calycosin-7-O-glucoside	C22H22O10	447.1284	10	5	0.59	✔
21	Glycitin	C22H22O10	447.1284	10	5	0.36	✔
22	Scutellarein	C15H10O6	287.0553	6	4	2.2	✔
23	Liquiritin	C21H22O9	419.1337	9	5	0.41	✔
24	Ononin	C22H22O9	431.1337	9	4	1.31	✔
25	3-Hydroxy-9,10-dimethoxypterocarpan	C17H16O5	301.107	5	1	2.55	✔
26	Calycosin	C16H12O5	285.0755	5	2	2.38	✔
27	Isomucronulatol	C17H18O5	303.1228	5	2	2.63	✔
28	Isomucronulatol-7-O-glucoside	C23H28O10	465.1762	10	5	0.84	✔
29	Nodakenin	C20H24O9	409.1497	9	4	0.47	✔
30	Emodin	C15H10O5	271.06	5	3	3.01	✔
31	Chrysophanol	C15H10O4	255.065	4	2	3.54	✔
32	Cinnamic acid	C9H8O2	149.0596	2	1	1.91	✔
33	Naringenin	C15H12O5	273.0758	5	3	2.12	✔
34	Diosmetin	C16H12O6	301.0708	6	3	2.28	✔
35	Formononetin	C16H12O4	269.0808	4	1	3.1	✔
36	Histidine	C6H9N3O2	154.0623	5	4	-3	✔
37	Glutamic acid	C5H9NO4	146.0458	5	4	-3.25	✔
38	D-(+)-Mannose	C6H12O6	179.056	6	5	-2.64	✔
39	Citric acid	C6H8O7	191.02	7	4	-1.98	✔
40	Amber acid	C4H6O4	117.0194	4	2	-0.66	✔
41	Isoleucine	C6H13NO2	130.0874	3	3	-1.41	✔
42	Gallic acid	C7H6O5	169.0144	5	4	0.59	✔
43	Protocatechuic acid	C7H6O4	153.0193	4	3	0.88	✔
44	Hydroxytyrosol	C8H10O3	153.0558	3	3	0.52	✔
45	L-Tryptophan	C11H12N2O2	203.0829	4	4	-1.08	✔
46	Esculetin	C9H6O4	177.0196	4	2	1.02	✔
47	Caffeic acid	C9H8O4	179.0351	4	3	0.94	✔
48	7-Hydroxycoumarin	C9H6O3	161.0244	3	1	1.51	✔
49	p-Coumaric acid	C9H8O3	163.0403	3	2	1.43	✔
50	Isoferulic acid	C10H10O4	193.051	4	2	1.25	✔
51	Rosmarinic acid	C18H16O8	359.0776	8	5	1.63	✔
52	Eriodictyol	C15H12O6	287.0563	6	4	1.63	✔
53	Luteolin	C15H10O6	285.0408	6	4	1.97	✔
54	Quercetin	C15H10O7	301.0357	7	5	1.68	✔
55	Vanillic acid	C8H8O4	167.0352	4	2	1.19	✔
56	Aurantio-obtusin	C17H14O7	329.0669	7	3	3.01	✔
57	Rhein	C15H8O6	283.0251	6	3	3	✔
58	Asiatic acid	C30H48O5	487.3433	5	4	4.7	✔

## Data Availability

The data used to support the findings of this study are available from the corresponding author upon request.

## Abbreviations

AKI: Acute kidney injury
XXD: Xiaoyu Xiezhuo Drink
UHPLC-Q-TOF-MS: Ultra-high-performance liquid chromatography coupled with hybrid triple quadrupole time-of-flight mass spectrometry
PPI: Protein-protein interaction
I/R: Ischemia-reperfusion
ARF: Acute renal failure
GFR: Glomerular filtration rate
CKD: Chronic kidney disease
IL-6: Interleukin 6
TNF-*α*: Tumor necrosis factor *α*
ICAM-1: Intercellular adhesion molecule 1
TGF-*β*: Transforming growth factor *β*
MMP-7/9: Metalloproteinase 7/9
TCM: Traditional Chinese medicine
*α*-SMA: *α*-Smooth muscle actin
UUO: Unilateral ureteral occlusion.

## Supplementary Materials

Supplementary 1 Table S1: high-resolution mass spectrometry data and elemental composition of XXD (positive ion mode).

Supplementary 2 Table S2: high-resolution mass spectrometry data and elemental composition of XXD (negative ion mode).
