# Supplementary material for: Xiaoyu Xiezhuo Drink Protects against Ischemia-Reperfusion Acute Kidney Injury in Aged Mice through Inhibiting the TGF-β1/Smad3 and HIF1 Signaling Pathways
Source: Biomed Res Int. 2021 Sep 9;2021:9963732. doi: 10.1155/2021/9963732 (PMC8449228; doi:10.1155/2021/9963732)
Supplement: Supplementary 1 — Table S1: high-resolution mass spectrometry data and elemental composition of XXD (positive ion mode). [file 9963732.f1.doc]

**Table S1 High-resolution mass spectrometry data and elemental composition of XYXZD (Positive Ion Mode)**

| No. | Component Name | Area | Retention Time | Formula | Precursor Mass | Found At Mass | Mass Error (ppm) | Library Score | Isotope Ratio Difference | nON | nOHNH | miLogP | Results | Canonical SMILES |
| --- | --- | --- | --- | --- | --- | --- | --- | --- | --- | --- | --- | --- | --- | --- |
| 1 | L(+)-Arginine | 9.33E+06 | 1.48 | C6H14N4O2 | 175.119 | 175.1188 | -0.8 | 86.3 | 1.3 | 6 | 7 | -3.76 | ✘ | C(CC(C(=O)O)N)CN=C(N)N |
| 2 | Betaine | 2.89E+07 | 1.53 | C5H11NO2 | 118.086 | 118.0861 | -1.1 | 98.6 | 0.3 | 3 | 0 | -5.41 | ✔ | C[N+](C)(C)CC(=O)[O-] |
| 3 | Trigonelline | 3.41E+06 | 1.59 | C7H7NO2 | 138.055 | 138.0551 | 0.8 | 94.5 | 0.5 | 3 | 0 | -5.40 | ✔ | C[N+]1=CC=CC(=C1)C(=O)[O-] |
| 4 | Proline | 1.85E+07 | 1.63 | C5H9NO2 | 116.071 | 116.0706 | -0.4 | 83.3 | 1.2 | 3 | 2 | -1.72 | ✔ | C1CC(NC1)C(=O)O |
| 5 | Stachydrine | 3.12E+06 | 1.67 | C7H13NO2 | 144.102 | 144.1018 | -0.6 | 98.7 | 0.7 | 3 | 0 | -5.31 | ✔ | C[N+]1(CCCC1C(=O)[O-])C |
| 6 | Adenine | 9.07E+06 | 2.14 | C5H5N5 | 136.062 | 136.0616 | -1.2 | 98.8 | 0.5 | 5 | 3 | 0.23 | ✔ | C1=NC2=NC=NC(=C2N1)N |
| 7 | Cytidine | 2.81E+05 | 2.27 | C9H13N3O5 | 244.093 | 244.093 | 1 | 100 | 1.7 | 8 | 5 | -1.93 | ✔ | C1=CN(C(=O)N=C1N)C2C(C(C(O2)CO)O)O |
| 8 | Nicotinic acid | 3.46E+06 | 2.32 | C6H5NO2 | 124.039 | 124.0391 | -1.7 | 99.8 | 0 | 3 | 1 | 0.27 | ✔ | C1=CC(=CN=C1)C(=O)O |
| 9 | Nicotinamide | 8.73E+05 | 2.5 | C6H6N2O | 123.055 | 123.0552 | -0.7 | 98.6 | 0.5 | 3 | 2 | -0.48 | ✔ | C1=CC(=CN=C1)C(=O)N |
| 10 | Adenosine | 8.99E+06 | 3.61 | C10H13N5O4 | 268.104 | 268.1038 | -0.7 | 100 | 2.7 | 9 | 5 | -0.85 | ✔ | C1=NC(=C2C(=N1)N(C=N2)C3C(C(C(O3)CO)O)O)N |
| 11 | Guanosine | 2.01E+06 | 3.87 | C10H13N5O5 | 284.099 | 284.0992 | 0.8 | 100 | 0.9 | 10 | 6 | -2.02 | ✘ | C1=NC2=C(N1C3C(C(C(O3)CO)O)O)N=C(NC2=O)N |
| 12 | 6-Hydroxypurine | 4.79E+06 | 3.91 | C5H4N4O | 137.046 | 137.0457 | -0.9 | 98.8 | 1 | 5 | 2 | -0.73 | ✔ | C1=NC2=C(N1)C(=O)NC=N2 |
| 13 | Phenylalanine | 6.58E+06 | 5.15 | C9H11NO2 | 166.086 | 166.0862 | -0.4 | 97.9 | 0.1 | 3 | 3 | -1.23 | ✔ | C1=CC=C(C=C1)CC(C(=O)O)N |
| 14 | Higenamine | 8.34E+04 | 7.7 | C16H17NO3 | 272.128 | 272.1284 | 0.9 | 83.6 | 1.5 | 4 | 4 | 2.00 | ✔ | C1CNC(C2=CC(=C(C=C21)O)O)CC3=CC=C(C=C3)O |
| 15 | Complanatoside | 1.24E+05 | 8.18 | C28H32O16 | 625.176 | 625.1769 | 0.9 | 100 | 4.1 | 16 | 9 | -1.13 | ✘ | COC1=CC(=C2C(=C1)OC(=C(C2=O)OC3C(C(C(C(O3)CO)O)O)O)C4=CC=C(C=C4)OC5C(C(C(C(O5)CO)O)O)O)O |
| 16 | 4-Hydroxybenzoic acid | 1.90E+05 | 8.34 | C7H6O3 | 139.039 | 139.0389 | -0.8 | 89 | 0.5 | 3 | 2 | 1.37 | ✔ | C1=CC(=CC=C1C(=O)O)O |
| 17 | Deacetyl asperulosidic acid methyl ester | 1.19E+05 | 8.62 | C17H24O11 | 422.166 | 422.1662 | 1.1 | 84.8 | 3.5 | 11 | 6 | -2.45 | ✘ | COC(=O)C1=COC(C2C1C(C=C2CO)O)OC3C(C(C(C(O3)CO)O)O)O |
| 18 | Gardenoside | 1.19E+05 | 8.62 | C17H24O11 | 422.166 | 422.1662 | 1.1 | 83.3 | 3.5 | 11 | 6 | -2.31 | ✘ | COC(=O)C1=COC(C2C1C=CC2(CO)O)OC3C(C(C(C(O3)CO)O)O)O |
| 19 | Catechin | 4.59E+05 | 8.84 | C15H14O6 | 291.086 | 291.0866 | 0.9 | 97.8 | 0.6 | 6 | 5 | 1.37 | ✔ | C1C(C(OC2=CC(=CC(=C21)O)O)C3=CC(=C(C=C3)O)O)O |
| 20 | Epicatechin | 4.59E+05 | 8.84 | C15H14O6 | 291.086 | 291.0866 | 0.9 | 97.1 | 0.6 | 6 | 5 | 1.37 | ✔ | C1C(C(OC2=CC(=CC(=C21)O)O)C3=CC(=C(C=C3)O)O)O |
| 21 | Daphnetin | 2.57E+05 | 9.53 | C9H6O4 | 179.034 | 179.0339 | 0 | 76 | 0.7 | 4 | 2 | 1.25 | ✔ | C1=CC(=C(C2=C1C=CC(=O)O2)O)O |
| 22 | Vitamin B2 | 8.40E+05 | 10.57 | C17H20N4O6 | 377.146 | 377.1458 | 0.5 | 99.5 | 2 | 10 | 5 | -0.76 | ✔ | CC1=CC2=C(C=C1C)N(C3=NC(=O)NC(=O)C3=N2)CC(C(C(CO)O)O)O |
| 23 | Polydatin | 1.65E+05 | 11.3 | C20H22O8 | 391.139 | 391.1391 | 1 | 91 | 1.7 | 8 | 6 | 1.20 | ✘ | C1=CC(=CC=C1C=CC2=CC(=CC(=C2)OC3C(C(C(C(O3)CO)O)O)O)O)O |
| 24 | Schaftoside | 1.71E+05 | 11.49 | C26H28O14 | 565.155 | 565.1556 | 0.8 | 97.2 | 2.1 | 14 | 10 | -1.68 | ✘ | C1C(C(C(C(O1)C2=C3C(=C(C(=C2O)C4C(C(C(C(O4)CO)O)O)O)O)C(=O)C=C(O3)C5=CC=C(C=C5)O)O)O)O |
| 25 | Kaempferol-3-gentiobioside | 8.54E+04 | 11.49 | C27H30O16 | 611.161 | 611.1616 | 1.5 | 75.8 | 2.8 | 16 | 10 | -1.58 | ✘ | C1=CC(=CC=C1C2=C(C(=O)C3=C(C=C(C=C3O2)O)O)OC4C(C(C(C(O4)COC5C(C(C(C(O5)CO)O)O)O)O)O)O)O |
| 26 | Geniposide | 1.24E+05 | 11.7 | C17H24O10.NH3 | 406.171 | 406.1711 | 0.8 | 95.2 | 3.4 | 10 | 5 | -1.53 | ✔ | COC(=O)C1=COC(C2C1CC=C2CO)OC3C(C(C(C(O3)CO)O)O)O |
| 27 | Hyperin | 1.13E+06 | 11.93 | C21H20O12 | 465.103 | 465.103 | 0.6 | 100 | 1.6 | 12 | 8 | -0.36 | ✘ | C1=CC(=C(C=C1C2=C(C(=O)C3=C(C=C(C=C3O2)O)O)OC4C(C(C(C(O4)CO)O)O)O)O)O |
| 28 | Syringaldehyde | 6.27E+04 | 12.51 | C9H10O4 | 183.065 | 183.0652 | 0.3 | 90.9 | 1.7 | 4 | 1 | 1.08 | ✔ | COC1=CC(=CC(=C1O)OC)C=O |
| 29 | Calycosin-7-O-glucoside | 1.14E+07 | 12.85 | C22H22O10 | 447.129 | 447.1284 | -0.3 | 98.3 | 1.2 | 10 | 5 | 0.59 | ✔ | COC1=C(C=C(C=C1)C2=COC3=C(C2=O)C=CC(=C3)OC4C(C(C(C(O4)CO)O)O)O)O |
| 30 | Glycitin | 1.14E+07 | 12.85 | C22H22O10 | 447.129 | 447.1284 | -0.3 | 97 | 1.2 | 10 | 5 | 0.36 | ✔ | COC1=C(C=C2C(=C1)C(=O)C(=CO2)C3=CC=C(C=C3)O)OC4C(C(C(C(O4)CO)O)O)O |
| 31 | Luteoloside | 6.76E+06 | 13.22 | C21H20O11 | 449.108 | 449.108 | 0.4 | 100 | 2.7 | 11 | 7 | 0.19 | ✘ | C1=CC(=C(C=C1C2=CC(=O)C3=C(C=C(C=C3O2)OC4C(C(C(C(O4)CO)O)O)O)O)O)O |
| 32 | Scutellarein | 1.01E+06 | 13.22 | C15H10O6 | 287.055 | 287.0553 | 1 | 93.3 | 1.8 | 6 | 4 | 2.20 | ✔ | C1=CC(=CC=C1C2=CC(=O)C3=C(O2)C=C(C(=C3O)O)O)O |
| 33 | Scutellarin | 4.57E+06 | 13.38 | C21H18O12 | 463.087 | 463.0873 | 0.3 | 99.5 | 2.1 | 12 | 7 | 0.07 | ✘ | C1=CC(=CC=C1C2=CC(=O)C3=C(C(=C(C=C3O2)OC4C(C(C(C(O4)C(=O)O)O)O)O)O)O)O |
| 34 | Acteoside | 8.64E+05 | 13.43 | C29H36O15 | 642.239 | 642.2399 | 1 | 98.4 | 1.9 | 15 | 9 | -0.45 | ✘ | CC1C(C(C(C(O1)OC2C(C(OC(C2OC(=O)C=CC3=CC(=C(C=C3)O)O)CO)OCCC4=CC(=C(C=C4)O)O)O)O)O)O |
| 35 | Liquiritin | 1.26E+06 | 13.72 | C21H22O9 | 419.134 | 419.1337 | 0.1 | 99.4 | 2.1 | 9 | 5 | 0.41 | ✔ | C1C(OC2=C(C1=O)C=CC(=C2)O)C3=CC=C(C=C3)OC4C(C(C(C(O4)CO)O)O)O |
| 36 | Genistin | 2.96E+05 | 14.67 | C21H20O10 | 433.113 | 433.1132 | 0.6 | 99.5 | 0.9 | 10 | 6 | 0.48 | ✘ | C1=CC(=CC=C1C2=COC3=CC(=CC(=C3C2=O)O)OC4C(C(C(C(O4)CO)O)O)O)O |
| 37 | Apigenin 7-O-beta-D-glucuronide | 3.17E+06 | 14.82 | C21H18O11 | 447.092 | 447.0926 | 0.9 | 100 | 2.2 | 11 | 6 | 0.55 | ✘ | C1=CC(=CC=C1C2=CC(=O)C3=C(C=C(C=C3O2)OC4C(C(C(C(O4)C(=O)O)O)O)O)O)O |
| 38 | Pratensein-7-O-glucoside | 7.99E+05 | 15.06 | C22H22O11 | 463.123 | 463.1239 | 0.8 | 95.3 | 2.8 | 11 | 6 | 0.30 | ✘ | COC1=C(C=C(C=C1)C2=COC3=CC(=CC(=C3C2=O)O)OC4C(C(C(C(O4)CO)O)O)O)O |
| 39 | Ononin | 6.96E+06 | 16.45 | C22H22O9 | 431.134 | 431.1337 | 0.1 | 98.6 | 0.4 | 9 | 4 | 1.31 | ✔ | COC1=CC=C(C=C1)C2=COC3=C(C2=O)C=CC(=C3)OC4C(C(C(C(O4)CO)O)O)O |
| 40 | Linarin | 3.51E+05 | 17.28 | C28H32O14 | 593.186 | 593.187 | 0.9 | 99.6 | 0.2 | 14 | 7 | 0.51 | ✘ | CC1C(C(C(C(O1)OCC2C(C(C(C(O2)OC3=CC(=C4C(=C3)OC(=CC4=O)C5=CC=C(C=C5)OC)O)O)O)O)O)O)O |
| 41 | 3-Hydroxy-9,10-Dimethoxypterocarpan | 4.39E+06 | 17.32 | C17H16O5 | 301.107 | 301.107 | -0.1 | 91.4 | 0.4 | 5 | 1 | 2.55 | ✔ | COC1=C(C2=C(C=C1)C3COC4=C(C3O2)C=CC(=C4)O)OC |
| 42 | Calycosin | 7.35E+06 | 17.61 | C16H12O5 | 285.076 | 285.0755 | -0.8 | 98.6 | 0.1 | 5 | 2 | 2.38 | ✔ | COC1=C(C=C(C=C1)C2=COC3=C(C2=O)C=CC(=C3)O)O |
| 43 | Isomucronulatol | 8.03E+05 | 17.73 | C17H18O5 | 303.123 | 303.1228 | 0.3 | 97.3 | 1.3 | 5 | 2 | 2.63 | ✔ | COC1=C(C(=C(C=C1)C2CC3=C(C=C(C=C3)O)OC2)O)OC |
| 44 | Isomucronulatol-7-O-glucoside | 3.24E+05 | 17.73 | C23H28O10 | 465.176 | 465.1762 | 1.4 | 77 | 0.9 | 10 | 5 | 0.84 | ✔ | COC1=C(C(=C(C=C1)C2CC3=C(C=C(C=C3)OC4C(C(C(C(O4)CO)O)O)O)OC2)O)OC |
| 45 | Nodakenin | 4.83E+05 | 17.98 | C20H24O9 | 409.149 | 409.1497 | 0.9 | 82.7 | 1.6 | 9 | 4 | 0.47 | ✔ | CC(C)(C1CC2=C(O1)C=C3C(=C2)C=CC(=O)O3)OC4C(C(C(C(O4)CO)O)O)O |
| 46 | Emodin | 2.63E+06 | 18.2 | C15H10O5 | 271.06 | 271.06 | -0.2 | 99.4 | 1 | 5 | 3 | 3.01 | ✔ | CC1=CC2=C(C(=C1)O)C(=O)C3=C(C2=O)C=C(C=C3O)O |
| 47 | Chrysophanol | 3.29E+06 | 18.42 | C15H10O4 | 255.065 | 255.065 | -0.6 | 97.2 | 0.7 | 4 | 2 | 3.54 | ✔ | CC1=CC2=C(C(=C1)O)C(=O)C3=C(C2=O)C=CC=C3O |
| 48 | Cinnamic acid | 1.08E+06 | 18.49 | C9H8O2 | 149.06 | 149.0596 | -0.6 | 91.2 | 1.2 | 2 | 1 | 1.91 | ✔ | C1=CC=C(C=C1)C=CC(=O)O |
| 49 | Naringenin | 1.25E+05 | 19.21 | C15H12O5 | 273.076 | 273.0758 | 0.2 | 96.2 | 1.3 | 5 | 3 | 2.12 | ✔ | C1C(OC2=CC(=CC(=C2C1=O)O)O)C3=CC=C(C=C3)O |
| 50 | Diosmetin | 2.24E+05 | 19.57 | C16H12O6 | 301.071 | 301.0708 | 0.4 | 92.2 | 1.6 | 6 | 3 | 2.28 | ✔ | COC1=C(C=C(C=C1)C2=CC(=O)C3=C(C=C(C=C3O2)O)O)O |
| 51 | Astragaloside Ⅳ | 1.03E+05 | 21.33 | C41H68O14 | 785.468 | 785.4691 | 1.2 | 92.6 | 2.1 | 14 | 9 | 1.21 | ✘ | CC1(C(CCC23C1C(CC4C2(C3)CCC5(C4(CC(C5C6(CCC(O6)C(C)(C)O)C)O)C)C)OC7C(C(C(C(O7)CO)O)O)O)OC8C(C(C(CO8)O)O)O)C |
| 52 | Formononetin | 4.26E+06 | 21.42 | C16H12O4 | 269.081 | 269.0808 | -0.1 | 99 | 0.1 | 4 | 1 | 3.10 | ✔ | COC1=CC=C(C=C1)C2=COC3=C(C2=O)C=CC(=C3)O |
| 53 | Glycyrrhetinic acid | 1.76E+05 | 26.34 | C30H46O4 | 471.347 | 471.3475 | 1.4 | 76 | 2.9 | 4 | 2 | 5.62 | ✘ | CC1(C2CCC3(C(C2(CCC1O)C)C(=O)C=C4C3(CCC5(C4CC(CC5)(C)C(=O)O)C)C)C)C |
| 54 | Betulonicacid | 3.82E+05 | 27.92 | C30H46O3 | 455.352 | 455.3523 | 0.7 | 72.9 | 0.9 | 3 | 1 | 6.86 | ✘ | CC(=C)C1CCC2(C1C3CCC4C5(CCC(=O)C(C5CCC4(C3(CC2)C)C)(C)C)C)C(=O)O |
| 55 | Ursolic Acid | 1.08E+05 | 28.34 | C30H48O3 | 457.368 | 457.3675 | -0.2 | 79.9 | 4.4 | 3 | 2 | 6.79 | ✘ | CC1CCC2(CCC3(C(=CCC4C3(CCC5C4(CCC(C5(C)C)O)C)C)C2C1C)C)C(=O)O |
