# Supplementary material for: Xiaoyu Xiezhuo Drink Protects against Ischemia-Reperfusion Acute Kidney Injury in Aged Mice through Inhibiting the TGF-β1/Smad3 and HIF1 Signaling Pathways
Source: Biomed Res Int. 2021 Sep 9;2021:9963732. doi: 10.1155/2021/9963732 (PMC8449228; doi:10.1155/2021/9963732)
Supplement: Supplementary 2 — Table S2: high-resolution mass spectrometry data and elemental composition of XXD (negative ion mode). [file 9963732.f2.doc]

**Table S2 High-resolution mass spectrometry data and elemental composition of XYXZD (Negative Ion Mode)**

| NO. | Component Name | Area | Retention Time | Formula | Precursor Mass | Found At Mass | Mass Error (ppm) | Library Score | Isotope Ratio Difference | nON | nOHNH | miLogP | Results | Canonical SMILES |
| --- | --- | --- | --- | --- | --- | --- | --- | --- | --- | --- | --- | --- | --- | --- |
| 1 | Histidine | 2.00E+05 | 1.36 | C6H9N3O2 | 154.062 | 154.0623 | 0.4 | 89.4 | 1.5 | 5 | 4 | -3.00 | ✔ | C1=C(NC=N1)CC(C(=O)O)N |
| 2 | Glutamic acid | 1.27E+05 | 1.4 | C5H9NO4 | 146.046 | 146.0458 | -0.3 | 97.9 | 0.4 | 5 | 4 | -3.25 | ✔ | C(CC(=O)O)C(C(=O)O)N |
| 3 | Sorbitol | 1.47E+06 | 1.42 | C6H14O6 | 181.072 | 181.0718 | 0 | 82.7 | 0.1 | 6 | 6 | -3.10 | ✘ | C(C(C(C(C(CO)O)O)O)O)O |
| 4 | D-(+)-Mannose | 1.15E+06 | 1.54 | C6H12O6 | 179.056 | 179.056 | -0.4 | 71.7 | 1.5 | 6 | 5 | -2.64 | ✔ | C(C1C(C(C(C(O1)O)O)O)O)O |
| 5 | Citric acid | 7.29E+06 | 2.45 | C6H8O7 | 191.02 | 191.02 | 1.4 | 97.6 | 0.7 | 7 | 4 | -1.98 | ✔ | C(C(=O)O)C(CC(=O)O)(C(=O)O)O |
| 6 | Amber Acid | 7.15E+05 | 3.05 | C4H6O4 | 117.019 | 117.0194 | 0.2 | 96.6 | 0.8 | 4 | 2 | -0.66 | ✔ | C(CC(=O)O)C(=O)O |
| 7 | Isoleucine | 6.07E+05 | 3.35 | C6H13NO2 | 130.087 | 130.0874 | 0.7 | 93.6 | 0.4 | 3 | 3 | -1.41 | ✔ | CCC(C)C(C(=O)O)N |
| 8 | Adenine | 3.10E+05 | 3.42 | C5H5N5 | 134.047 | 134.0473 | 0.9 | 94.7 | 1.6 | 5 | 3 | 0.23 | ✔ | C1=NC2=NC=NC(=C2N1)N |
| 9 | Guanosine | 7.13E+05 | 3.66 | C10H13N5O5 | 282.084 | 282.0847 | 1 | 99.4 | 0.9 | 10 | 6 | -2.02 | ✘ | C1=NC2=C(N1C3C(C(C(O3)CO)O)O)N=C(NC2=O)N |
| 10 | Gallic acid | 9.04E+06 | 3.7 | C7H6O5 | 169.014 | 169.0144 | 1 | 99.4 | 0.3 | 5 | 4 | 0.59 | ✔ | C1=C(C=C(C(=C1O)O)O)C(=O)O |
| 11 | 10-Deacetylasperulosidic acid | 1.06E+06 | 4.64 | C16H22O11 | 389.109 | 389.1094 | 1.2 | 97.5 | 3.8 | 11 | 7 | -3.06 | ✘ | C1=C(C2C(C1O)C(=COC2OC3C(C(C(C(O3)CO)O)O)O)C(=O)O)CO |
| 12 | Phenprobamate | 1.11E+06 | 4.84 | C9H11NO2 | 164.072 | 164.0718 | 0.4 | 96.3 | 0.6 | 3 | 3 | -1.23 | ✔ | C1=CC=C(C=C1)CC(C(=O)O)N |
| 13 | Geniposidic acid | 1.53E+06 | 5.13 | C16H22O10 | 373.114 | 373.1145 | 1.4 | 77.3 | 1.4 | 10 | 6 | -2.15 | ✘ | C1C=C(C2C1C(=COC2OC3C(C(C(C(O3)CO)O)O)O)C(=O)O)CO |
| 14 | Protocatechuic acid | 2.34E+05 | 5.84 | C7H6O4 | 153.019 | 153.0193 | -0.3 | 94.2 | 0.8 | 4 | 3 | 0.88 | ✔ | C1=CC(=C(C=C1C(=O)O)O)O |
| 15 | Hydroxytyrosol | 9.20E+04 | 6.17 | C8H10O3 | 153.056 | 153.0558 | 0.6 | 93 | 0.7 | 3 | 3 | 0.52 | ✔ | C1=CC(=C(C=C1CCO)O)O |
| 16 | L-Tryptophan | 1.63E+06 | 6.95 | C11H12N2O2 | 203.083 | 203.0829 | 1.3 | 92.4 | 1.6 | 4 | 4 | -1.08 | ✔ | C1=CC=C2C(=C1)C(=CN2)CC(C(=O)O)N |
| 17 | 8-Epiloganic acid | 1.16E+06 | 7.14 | C16H24O10 | 375.13 | 375.13 | 1 | 98.2 | 1 | 10 | 6 | -1.87 | ✘ | CC1C(CC2C1C(OC=C2C(=O)O)OC3C(C(C(C(O3)CO)O)O)O)O |
| 18 | Catechin | 8.48E+05 | 8.32 | C15H14O6 | 289.072 | 289.0721 | 1.2 | 96.1 | 1.6 | 6 | 5 | 1.37 | ✔ | C1C(C(OC2=CC(=CC(=C21)O)O)C3=CC(=C(C=C3)O)O)O |
| 19 | Chlorogenic acid | 7.32E+04 | 8.34 | C16H18O9 | 353.088 | 353.0882 | 1 | 95.1 | 3.3 | 9 | 6 | -0.45 | ✘ | C1C(C(C(CC1(C(=O)O)O)OC(=O)C=CC2=CC(=C(C=C2)O)O)O)O |
| 20 | Esculetin | 6.70E+05 | 8.96 | C9H6O4 | 177.019 | 177.0196 | 1.5 | 96.5 | 0.2 | 4 | 2 | 1.02 | ✔ | C1=CC(=O)OC2=CC(=C(C=C21)O)O |
| 21 | Caffeic acid | 1.41E+06 | 9.17 | C9H8O4 | 179.035 | 179.0351 | 0.9 | 92.8 | 0.1 | 4 | 3 | 0.94 | ✔ | C1=CC(=C(C=C1C=CC(=O)O)O)O |
| 22 | 7-Hydroxycoumarin | 1.41E+04 | 9.94 | C9H6O3 | 161.024 | 161.0244 | 0 | 84.8 | 3.5 | 3 | 1 | 1.51 | ✔ | C1=CC(=CC2=C1C=CC(=O)O2)O |
| 23 | Isoschaftoside | 1.85E+05 | 10.85 | C26H28O14 | 563.141 | 563.1416 | 1.7 | 98.6 | 2.5 | 14 | 10 | -1.68 | ✘ | C1C(C(C(C(O1)C2=C(C(=C3C(=C2O)C(=O)C=C(O3)C4=CC=C(C=C4)O)C5C(C(C(C(O5)CO)O)O)O)O)O)O)O |
| 24 | Hyperin | 1.70E+06 | 11.27 | C21H20O12 | 463.088 | 463.0891 | 1.9 | 94.2 | 0.7 | 12 | 8 | -0.36 | ✘ | C1=CC(=C(C=C1C2=C(C(=O)C3=C(C=C(C=C3O2)O)O)OC4C(C(C(C(O4)CO)O)O)O)O)O |
| 25 | p-Coumaric acid | 4.72E+05 | 11.3 | C9H8O3 | 163.04 | 163.0403 | 1.5 | 97.6 | 0.7 | 3 | 2 | 1.43 | ✔ | C1=CC(=CC=C1C=CC(=O)O)O |
| 26 | leutheroside E | 5.31E+04 | 11.38 | C34H46O18 | 787.267 | 787.2669 | 0.4 | 98.5 | 4.7 | 18 | 8 | -1.48 | ✘ | COC1=CC(=CC(=C1OC2C(C(C(C(O2)CO)O)O)O)OC)C3C4COC(C4CO3)C5=CC(=C(C(=C5)OC)OC6C(C(C(C(O6)CO)O)O)O)OC |
| 27 | Plantamajoside | 1.58E+07 | 12.02 | C29H36O16 | 639.193 | 639.1942 | 1.8 | 92.2 | 1.8 | 16 | 10 | -1.46 | ✘ | C1=CC(=C(C=C1CCOC2C(C(C(C(O2)CO)OC(=O)C=CC3=CC(=C(C=C3)O)O)OC4C(C(C(C(O4)CO)O)O)O)O)O)O |
| 28 | Calycosin-7-o-glucoside | 6.69E+06 | 12.16 | C22H22O10 | 491.119 | 491.1202 | 1.5 | 99.5 | 1.7 | 10 | 5 | 0.59 | ✔ | COC1=C(C=C(C=C1)C2=COC3=C(C2=O)C=CC(=C3)OC4C(C(C(C(O4)CO)O)O)O)O |
| 29 | Isoferulic acid | 1.43E+05 | 12.3 | C10H10O4 | 193.051 | 193.051 | 2 | 97.4 | 2.5 | 4 | 2 | 1.25 | ✔ | COC1=C(C=C(C=C1)C=CC(=O)O)O |
| 30 | (-)-Catechin Gallate | 6.85E+05 | 12.44 | C22H18O10 | 441.083 | 441.0831 | 1 | 93.5 | 2.6 | 10 | 7 | 2.54 | ✘ | C1C(C(OC2=CC(=CC(=C21)O)O)C3=CC(=C(C=C3)O)O)OC(=O)C4=CC(=C(C(=C4)O)O)O |
| 31 | Forsythoside B | 1.83E+05 | 12.45 | C34H44O19 | 755.24 | 755.2412 | 1 | 98.7 | 1.8 | 19 | 11 | -1.86 | ✘ | CC1C(C(C(C(O1)OC2C(C(OC(C2OC(=O)C=CC3=CC(=C(C=C3)O)O)COC4C(C(CO4)(CO)O)O)OCCC5=CC(=C(C=C5)O)O)O)O)O)O |
| 32 | Luteoloside | 8.35E+06 | 12.53 | C21H20O11 | 447.093 | 447.094 | 1.5 | 98.7 | 0.4 | 11 | 7 | 0.19 | ✘ | C1=CC(=C(C=C1C2=CC(=O)C3=C(C=C(C=C3O2)OC4C(C(C(C(O4)CO)O)O)O)O)O)O |
| 33 | Scutellarin | 3.91E+06 | 12.68 | C21H18O12 | 461.073 | 461.0733 | 1.6 | 100 | 0.9 | 12 | 7 | 0.07 | ✘ | C1=CC(=CC=C1C2=CC(=O)C3=C(C(=C(C=C3O2)OC4C(C(C(C(O4)C(=O)O)O)O)O)O)O)O |
| 34 | Verbascoside | 3.78E+07 | 12.75 | C29H36O15 | 623.198 | 623.199 | 1.4 | 98.5 | 4.1 | 15 | 9 | -0.45 | ✘ | CC1C(C(C(C(O1)OC2C(C(OC(C2OC(=O)C=CC3=CC(=C(C=C3)O)O)CO)OCCC4=CC(=C(C=C4)O)O)O)O)O)O |
| 35 | Calceorioside B | 5.82E+06 | 12.91 | C23H26O11 | 477.14 | 477.1407 | 1 | 92.4 | 2 | 11 | 7 | 0.69 | ✘ | C1=CC(=C(C=C1CCOC2C(C(C(C(O2)COC(=O)C=CC3=CC(=C(C=C3)O)O)O)O)O)O)O |
| 36 | Aempferol-3-O-rutinoside | 4.29E+05 | 13.06 | C27H30O15 | 593.151 | 593.1518 | 1 | 98 | 0.3 | 15 | 9 | -1.64 | ✘ | [C@H]1(O[C@H]([C@@H]([C@@H]([C@H]1O)O)O)OC[C@@H]1[C@H]([C@@H]([C@H]([C@@H](O1)OC1=C(OC2C(=C(C=C(C2)O)O)C1=O)c1cc(ccc1)O)O)O)O)C |
| 37 | Narcissoside | 9.31E+04 | 13.27 | C28H32O16 | 623.162 | 623.1633 | 2.4 | 83.6 | 4.6 | 16 | 9 | -0.76 | ✘ | CC1C(C(C(C(O1)OCC2C(C(C(C(O2)OC3=C(OC4=CC(=CC(=C4C3=O)O)O)C5=CC(=C(C=C5)O)OC)O)O)O)O)O)O |
| 38 | Cyasterone | 3.25E+05 | 14.17 | C29H44O8 | 519.296 | 519.2966 | 0.5 | 95.5 | 3.3 | 8 | 5 | 0.71 | ✘ | CC1C(C(OC1=O)C)CC(C(C)(C2CCC3(C2(CCC4C3=CC(=O)C5C4(CC(C(C5)O)O)C)C)O)O)O |
| 39 | Rosmarinic acid | 1.77E+05 | 14.49 | C18H16O8 | 359.077 | 359.0776 | 1 | 97.3 | 2.4 | 8 | 5 | 1.63 | ✔ | C1=CC(=C(C=C1CC(C(=O)O)OC(=O)C=CC2=CC(=C(C=C2)O)O)O)O |
| 40 | 4-Hydroxybenzoic acid | 8.33E+05 | 14.85 | C7H6O3 | 137.024 | 137.0244 | 0.2 | 100 | 0.4 | 3 | 2 | 1.37 | ✔ | C1=CC(=CC=C1C(=O)O)O |
| 41 | Phloridzin | 3.81E+05 | 14.9 | C21H24O10 | 435.13 | 435.13 | 0.8 | 99 | 2.9 | 10 | 7 | 0.40 | ✘ | C1=CC(=CC=C1CCC(=O)C2=C(C=C(C=C2OC3C(C(C(C(O3)CO)O)O)O)O)O)O |
| 42 | Ononin | 4.72E+06 | 15.72 | C22H22O9 | 475.125 | 475.125 | 1 | 99.4 | 3.2 | 9 | 4 | 1.31 | ✔ | COC1=CC=C(C=C1)C2=COC3=C(C2=O)C=CC(=C3)OC4C(C(C(C(O4)CO)O)O)O |
| 43 | Cistanoside D | 2.61E+06 | 15.79 | C31H40O15 | 651.229 | 651.2304 | 1.5 | 97.1 | 1.9 | 15 | 7 | 0.17 | ✘ | CC1C(C(C(C(O1)OC2C(C(OC(C2OC(=O)C=CC3=CC(=C(C=C3)O)OC)CO)OCCC4=CC(=C(C=C4)O)OC)O)O)O)O |
| 44 | Eriodictyol | 1.11E+05 | 16.53 | C15H12O6 | 287.056 | 287.0563 | 0.7 | 97 | 2 | 6 | 4 | 1.63 | ✔ | C1C(OC2=CC(=CC(=C2C1=O)O)O)C3=CC(=C(C=C3)O)O |
| 45 | Luteolin | 1.40E+06 | 16.68 | C15H10O6 | 285.04 | 285.0408 | 1.2 | 97.5 | 2.2 | 6 | 4 | 1.97 | ✔ | C1=CC(=C(C=C1C2=CC(=O)C3=C(C=C(C=C3O2)O)O)O)O |
| 46 | Quercetin | 4.79E+04 | 16.75 | C15H10O7 | 301.035 | 301.0357 | 1 | 77 | 1.9 | 7 | 5 | 1.68 | ✔ | C1=CC(=C(C=C1C2=C(C(=O)C3=C(C=C(C=C3O2)O)O)O)O)O |
| 47 | Calycosin | 5.81E+06 | 16.85 | C16H12O5 | 283.061 | 283.0615 | 1.2 | 99.1 | 2 | 5 | 2 | 2.38 | ✔ | COC1=C(C=C(C=C1)C2=COC3=C(C2=O)C=CC(=C3)O)O |
| 48 | Isomucronulatol-7-O-glucoside | 3.71E+06 | 17.01 | C23H28O10 | 463.161 | 463.1614 | 1 | 98.1 | 2.7 | 10 | 5 | 0.84 | ✔ | COC1=C(C(=C(C=C1)C2CC3=C(C=C(C=C3)OC4C(C(C(C(O4)CO)O)O)O)OC2)O)OC |
| 49 | Emodin-8-glucoside | 1.23E+07 | 17.5 | C21H20O10 | 431.098 | 431.0989 | 1.3 | 94.3 | 0.8 | 10 | 6 | 0.96 | ✘ | CC1=CC2=C(C(=C1)O)C(=O)C3=C(C2=O)C=C(C=C3OC4C(C(C(C(O4)CO)O)O)O)O |
| 50 | Chrysophanol | 6.85E+06 | 17.7 | C15H10O4 | 253.051 | 253.0509 | 1.2 | 93.6 | 0.8 | 4 | 2 | 3.54 | ✔ | CC1=CC2=C(C(=C1)O)C(=O)C3=C(C2=O)C=CC=C3O |
| 51 | 3-Hydroxy-9,10-Dimethoxypterocarpan | 1.04E+06 | 17.75 | C17H16O5 | 299.092 | 299.0927 | 0.8 | 93 | 1 | 5 | 1 | 2.55 | ✔ | COC1=C(C2=C(C=C1)C3COC4=C(C3O2)C=CC(=C4)O)OC |
| 52 | Vanillic acid | 3.29E+05 | 17.9 | C8H8O4 | 167.035 | 167.0352 | 1.2 | 93 | 0.3 | 4 | 2 | 1.19 | ✔ | COC1=C(C=CC(=C1)C(=O)O)O |
| 53 | Naringenin | 2.01E+05 | 18.46 | C15H12O5 | 271.061 | 271.0614 | 0.7 | 96.9 | 1.1 | 5 | 3 | 2.12 | ✔ | C1C(OC2=CC(=CC(=C2C1=O)O)O)C3=CC=C(C=C3)O |
| 54 | Pedunculoside | 7.08E+04 | 18.61 | C36H58O10 | 695.401 | 695.4019 | 1 | 98 | 4.9 | 10 | 7 | 2.96 | ✘ | CC1CCC2(CCC3(C(=CCC4C3(CCC5C4(CCC(C5(C)CO)O)C)C)C2C1(C)O)C)C(=O)OC6C(C(C(C(O6)CO)O)O)O |
| 55 | Aurantio-Obtusin | 3.74E+04 | 18.69 | C17H14O7 | 329.067 | 329.0669 | 0.7 | 80.4 | 1.5 | 7 | 3 | 3.01 | ✔ | CC1=CC2=C(C(=C1O)OC)C(=O)C3=C(C(=C(C=C3C2=O)O)OC)O |
| 56 | Formononetin | 3.32E+06 | 20.67 | C16H12O4 | 267.066 | 267.0666 | 1.3 | 98.3 | 2 | 4 | 1 | 3.10 | ✔ | COC1=CC=C(C=C1)C2=COC3=C(C2=O)C=CC(=C3)O |
| 57 | Astragaloside Ⅳ | 8.51E+05 | 20.69 | C41H68O14 | 829.459 | 829.4599 | 0.9 | 100 | 0.2 | 14 | 9 | 1.21 | ✘ | CC1(C(CCC23C1C(CC4C2(C3)CCC5(C4(CC(C5C6(CCC(O6)C(C)(C)O)C)O)C)C)OC7C(C(C(C(O7)CO)O)O)O)OC8C(C(C(CO8)O)O)O)C |
| 58 | Chikusetsusponin Iva | 9.16E+04 | 21.09 | C42H66O14 | 793.438 | 793.4384 | 0.6 | 95.6 | 1.5 | 14 | 8 | 3.19 | ✘ | CC1(CCC2(CCC3(C(=CCC4C3(CCC5C4(CCC(C5(C)C)OC6C(C(C(C(O6)C(=O)O)O)O)O)C)C)C2C1)C)C(=O)OC7C(C(C(C(O7)CO)O)O)O)C |
| 59 | Rhein | 7.23E+06 | 22.72 | C15H8O6 | 283.025 | 283.0251 | 1 | 99 | 1.3 | 6 | 3 | 3.00 | ✔ | C1=CC2=C(C(=C1)O)C(=O)C3=C(C2=O)C=C(C=C3O)C(=O)O |
| 60 | Glycyrrhizic acid | 1.52E+04 | 22.91 | C42H62O16 | 821.397 | 821.3958 | -0.9 | 70.5 | 4.7 | 16 | 8 | 1.97 | ✘ | CC1(C2CCC3(C(C2(CCC1OC4C(C(C(C(O4)C(=O)O)O)O)OC5C(C(C(C(O5)C(=O)O)O)O)O)C)C(=O)C=C6C3(CCC7(C6CC(CC7)(C)C(=O)O)C)C)C)C |
| 61 | α-Hederin | 2.77E+04 | 23.09 | C41H66O12 | 795.454 | 795.4542 | 0.7 | 100 | 1.2 | 12 | 7 | 3.57 | ✘ | CC1C(C(C(C(O1)OC2C(C(COC2OC3CCC4(C(C3(C)CO)CCC5(C4CC=C6C5(CCC7(C6CC(CC7)(C)C)C(=O)O)C)C)C)O)O)O)O)O |
| 62 | Astragaloside I | 3.44E+06 | 23.85 | C45H72O16 | 913.48 | 913.4811 | 0.9 | 97.9 | 4.7 | 16 | 7 | 2.40 | ✘ | CC(=O)OC1C(COC(C1OC(=O)C)OC2CCC34CC35CCC6(C(C(CC6(C5CC(C4C2(C)C)OC7C(C(C(C(O7)CO)O)O)O)C)O)C8(CCC(O8)C(C)(C)O)C)C)O |
| 63 | Asiatic acid | 3.01E+05 | 24.2 | C30H48O5 | 487.343 | 487.3433 | 0.7 | 82.1 | 2.9 | 5 | 4 | 4.70 | ✔ | CC1CCC2(CCC3(C(=CCC4C3(CCC5C4(CC(C(C5(C)CO)O)O)C)C)C2C1C)C)C(=O)O |
| 64 | Emodin | 2.92E+06 | 24.8 | C15H10O5 | 269.046 | 269.046 | 1.5 | 98.3 | 0.8 | 5 | 3 | 3.01 | ✔ | CC1=CC2=C(C(=C1)O)C(=O)C3=C(C2=O)C=C(C=C3O)O |
| 65 | Gingerglycolipid B | 5.88E+04 | 25.75 | C33H58O14 | 723.381 | 723.3812 | 0.5 | 96.9 | 1.9 | 14 | 8 | 2.48 | ✘ | CCCCCC=CCC=CCCCCCCCC(=O)OCC(COC1C(C(C(C(O1)COC2C(C(C(C(O2)CO)O)O)O)O)O)O)O |
| 66 | Corosolic acid | 2.21E+05 | 25.92 | C30H48O4 | 471.348 | 471.3481 | 0.3 | 92.3 | 0.7 | 4 | 3 | 5.87 | ✘ | CC1CCC2(CCC3(C(=CCC4C3(CCC5C4(CC(C(C5(C)C)O)O)C)C)C2C1C)C)C(=O)O |
| 67 | Ursolic Acid | 1.18E+05 | 28.12 | C30H48O3 | 455.353 | 455.3531 | 0.2 | 100 | 1.2 | 3 | 2 | 6.79 | ✘ | CC1CCC2(CCC3(C(=CCC4C3(CCC5C4(CCC(C5(C)C)O)C)C)C2C1C)C)C(=O)O |
